# Peer advocacy and access to hospital care for people who are homeless in London, UK, 2019–2023: a cohort study

**DOI:** 10.1136/bmjopen-2025-107422

**Published:** 2026-07-16

**Authors:** Lucy Platt, Andrew Guise, Paniz Hosseini, Kate Bowgett, Martin Murphy, Mani Cudjoe, PJ Annand, Michael Spike Hudson, Maame Esi D Yankah, Dee Menezes, Rosa Legood, Rob W Aldridge, Andrew Hayward, Serena Luchenski, Elizabeth Williamson, Sujit D Rathod

**Affiliations:** 1 Faculty of Public Health and Policy, London School of Hygiene & Tropical Medicine, London, England, UK; 2 Population Health Sciences, King’s College London, London, UK; 3 Groundswell, London, UK; 4 Centre for Care, Social Research Institutes, The University of Sheffield Faculty of Social Sciences, Sheffield, England, UK; 5 Institute of Health Informatics Research, University College London, London, UK; 6 Institute of Epidemiology and Healthcare, University College London, London, UK; 7 Faculty of Epidemiology and Population Health, London School of Hygiene & Tropical Medicine, London, England, UK

**Keywords:** Peer Group, Health Services, Electronic Health Records

## Abstract

**Abstract:**

**Objectives:**

To measure differences in hospital use between homeless adults using the homeless health peer advocacy (HHPA) service (clients) and non-clients in London.

**Design:**

We conducted a cohort study with linkage to Hospital Episode Statistics (HES) 1 year prior and postenrolment.

**Setting:**

London, UK.

**Population:**

People who are homeless in London aged over 18 years residing in a hostel, attending a day centre or being referred by a homelessness service; experiencing difficulties accessing healthcare; and speaking either English or Polish. Participants were required to provide consent for linkage to HES. To be classified as a client, individuals must have used the HHPA service at least once between January and July 2021; non-clients were those who had never used the service.

**Intervention:**

Peer advocacy is the provision of support by volunteer-trained advocates with lived experience of homelessness to individuals to overcome barriers to accessing health services.

**Outcomes:**

The primary outcome was not attending a scheduled outpatient appointment (‘did-not-attend’) over 12 months postrecruitment, commencing from their baseline interview date. Secondary outcomes included the number of accident and emergency (A&E) and inpatient admissions (all and planned admissions) during that same period.

**Methods:**

We estimated the probability of non-attendance using Poisson regression and the number of inpatient admissions and A&E visits using negative binomial regression models. Models included: (1) propensity score weights and (2) propensity score weights and imbalanced confounders. Sensitivity analyses assumed that participants who did not link to HES had no hospital attendance. Exploratory analyses examined differential effects of peer advocacy by clients’ type of peer advocacy engagement (new vs ongoing clients; supported vs unsupported) and by clients’ anxiety or depression symptom scores measured with the Patient Health Questionnaire-4 (PHQ4).

**Results:**

153 clients and 158 non-clients were recruited between July and December 2021. Most were male (77.5%) with a median age of 48 years. Weighted regression models suggested no evidence of effect of peer advocacy on non-attendance (rate ratio (RR) 0.97 (95% CI 0.67 to 1.42)), no difference in the mean number of A&E visits (2.59 95% CI 1.93, 3.24 vs 1.76 95% CI 1.13, 2.40) but more inpatient admissions (1.65 95% CI 1.10, 2.20 vs. 0.53 95% CI 0.27, 0.82) for HHPA clients vs non-clients respectively. This was supported in sensitivity analyses. In exploratory analyses, clients with PHQ4 scores of 9–12 had greater probability of non-attendance at outpatient appointments (RR 1.98 (95% CI 1.0 to 3.89)) compared to non-clients. Those with scores of 6–8 had 5.86 (95% CI 2.73 to 9.0) completed appointments versus 1.87 (95% CI 0.41 to 3.34) among non-clients and 1.13 (95% CI 0.01 to 0.27) inpatient admissions compared with 0.13 (95% CI −0.01 to −0.27) among non-clients.

**Conclusions:**

Following COVID-related disruptions to the work of peer advocates and health services, we found mixed evidence on the effect of peer advocacy: with no evidence of impact on outpatient appointments or use of emergency services; but increased inpatient admissions.

**STRENGTHS AND LIMITATIONS OF THIS STUDY**

- Use of linked hospital data minimised recall bias by relying on objective outcome measures sourced from national Hospital Episode Statistics (HES), enhancing data accuracy and reliability.
- Application of propensity score weighting addressed known baseline imbalances between client and non-client groups, allowing more robust adjustment for confounding and strengthening causal inference in a non-randomised design.
- Potential for residual confounding remains, given notable differences between peer advocacy clients and non-clients and the possibility of unmeasured confounding.
- Data linkage was incomplete, with 26% of participants not matched to HES records, introducing potential selection bias and limiting confidence in sensitivity analyses that assumed non-attendance among unlinked individuals.
- Impact of COVID-19 disrupted both intervention fidelity and usual care, with reduced in-person peer support and hospital access potentially diluting the observed effects of the intervention and affecting generalisability.

## Introduction

 Homelessness includes sleeping outside, in hostels, shelters and temporary accommodation, as well as residing in unsafe and inadequate housing (eg, overcrowded conditions or settings involving domestic violence).^1^ In the UK, all forms of homelessness are increasing. In 2023, 3898 people were observed sleeping outside on a single night—a 27% increase from 2022—and this is thought to underestimate the true number.^2^ Local authorities estimated a 20% increase in households in temporary accommodation between 2018–2019 and 2022.^3^

Homeless organisations in England and Wales report that 14% of people experiencing homelessness have chronic respiratory disease, 17% have tuberculosis and 10% are living with hepatitis C infection. Overall, 80% report one or more chronic physical health problems.^4^ Self-reported mental health diagnoses increased from 45% in 2014 to 82% in 2022.^4 5^ Standardised mortality rates among marginalised populations—including those with experience of homelessness—are between 7.9 and 11.9 times higher for men and women compared with stably housed populations, and four times higher than populations in the most deprived areas.^6^

These inequalities in health reflect not only multiple experiences of exclusion and elevated health needs, but also limited access to healthcare. People experiencing homelessness have high rates of accident and emergency (A&E) use (50% at least once in the past year) and hospital admissions (38% in the last year).^7^ These high rates represent avoidable ill-health and distress, as well as considerable costs to the health system: healthcare costs for homeless individuals are estimated to be eight times higher than those of the general population.^7^

Peer advocacy involves training people with lived experience of homelessness, or who are otherwise marginalised due to drug use or mental health issues, to support and advocate for the services and rights of their peers—those with similar experiences—often within health and social care settings. Interventions such as peer advocacy can address several healthcare challenges, including overcoming barriers to accessing health services. The availability of a trusted advocate—with lived experience of homelessness—can reduce structural (eg, stigma or alienation, transport), social (eg, navigating interactions with staff) and bureaucratic (eg, paperwork) barriers to accessing healthcare.^8^,^12^ Evidence supports the effectiveness of community-delivered peer support to facilitate recovery from severe mental health conditions,^13^ as well as to support harm reduction, HIV prevention and treatment of tuberculosis and hepatitis C in the context of drug use.^14^,^16^ A recent feasibility study has demonstrated that provision of practical and emotional support by peer navigators to people using drugs and the homeless is acceptable and was linked to decreases in drug use and increases in uptake of opioid substitution therapy.^17^ Overlapping with this, there is an emerging evidence base highlighting the potential of peer interventions among sex workers, prison and homeless populations.^18^,^20^ However, peer interventions in the UK have historically received limited funding and lack national government backing.^1^ Moreover, a shortage of evaluations using well-theorised interventions and rigorous study designs means there is still insufficient evidence to support a more comprehensive roll-out.^13 21^

We conducted a mixed-methods evaluation of a peer advocacy intervention among homeless adults in London, assessing its impact, cost-consequences and processes related to hospital utilisation.^22^ This paper reports on findings of the impact evaluation that measured the extent to which a peer advocacy intervention increases access to hospital care.

## Methods

### Study design

We conducted a prospective cohort study of people experiencing homelessness who had difficulty meeting their healthcare needs: both peer advocacy clients and non-clients.

### Patient and public involvement

We used participatory approaches, involving people with lived experience of homelessness or those working with homeless individuals as co-researchers, to develop methods, gather data and conduct analysis. The evaluation was overseen by a steering group consisting of people working in the homeless sector and with lived experience of homelessness to guide the design and interpretation of findings.^22^ The intervention was delivered by Groundswell, a non-governmental organisation staffed by individuals with lived experience of homelessness, working alongside specialist homelessness services in London.

### Intervention and impact of COVID-19

We evaluated the Homeless Health Peer Advocacy (HHPA) service. HHPA supports people who are homeless and have difficulty managing their healthcare needs. HHPA includes assistance with healthcare appointments. Clients are generally aged over 25, although HHPA also works with individuals aged 18–24 years. Peers are individuals with lived experience of homelessness who work as volunteers and are reimbursed for expenses. They complete 6 weeks of training for the role outlined in a manual, followed by a 1 month shadowing period. The training includes risk assessment skills, safeguarding policies and managing difficult behaviour. Peers are supervised by salaried staff at Groundswell, who ensure that the role is carried out as specified. Staff also support service delivery by processing bookings from referral agencies to enable a timely and effective response, and by organising counselling for peers when needed. Initial meetings allow peers and clients to build rapport. Peer advocacy is not time-bound and may be open-ended where needed.^23^

Peer advocacy activities include accompaniment to healthcare appointments, assistance with general practitioner (GP) registration, one-on-one conversations and financial help with transport. Peers also signpost clients to other relevant services when needs fall outside HHPA’s remit (eg, nutrition, housing or legal advice). Clients may self-refer, be referred by a key worker, or be approached through peer ‘in-reach’ activities at hostels and day centres. HHPA is commissioned to operate in 10 of London’s 32 boroughs serving approximately 600 clients annually.

Recruitment took place between July 2021 and December 2021. During this period there were no lockdowns, but health services were still impacted by the COVID-19 pandemic. HHPA was providing less direct in-person advocacy, instead conducting welfare calls by phone, joining clients in online consultation for outpatient appointments and offering increased support for independent travel, documented in our linked qualitative work.^24^ Although 21 peer advocates were available, they were working less frequently. At the same time, the National Health Service (NHS) was experiencing significant delays in outpatient care and planned inpatient admissions and London’s outdoor sleeping population was provided emergency accommodation in hotels, where GP registration and other health and social care services were facilitated.^25^ Despite these disruptions and potential impacts on outcomes, the research team and steering group concluded that any changes would likely affect both clients and non-clients. Together we judged HHPA and NHS hospitals to be functioning sufficiently well to justify continuation of the evaluation.

### Eligibility criteria

Individuals aged over 18 years were eligible for the cohort study if they met the following criteria: residing in a hostel, attending a day centre or being referred by a homelessness service; experiencing difficulties accessing healthcare; and speaking either English or Polish. Participants were required to provide consent for linkage to Hospital Episode Statistics (HES). To be classified as a client, individuals must have used the HHPA service at least once between January and July 2021; non-clients were those who had never used the service. Clients were originally defined to be those scheduled for their first engagement with HHPA; the definition was expanded due to HHPA engaging fewer new clients as a result of COVID-19. Other deviations from the original protocol include our sample size calculation. This was initially based on an average number of outpatient non-attendances of 0.06 per year among HHPA clients and 0.42 for non-clients.^22^ We subsequently recalculated the sample size in line with our primary outcome. We estimated that HPPA clients will have 15% of scheduled outpatient appointments classified as non-attended in 12 months, compared with 34% for those in the comparison group based on previous evidence.^26^ With 90% power, a two-sided alpha of 0.05 and equal group sizes, the required analysis sample was 232 participants. Assuming that outcome data would be available for only 80% of participants via the NHS HES database, the recruitment target was set at 290.

### Recruitment

Clients were recruited by peer advocates, who were referred when an appointment with a peer advocate was made. Non-clients were recruited concurrently and opportunistically from hostels and day centres in areas of London where Groundswell was not commissioned to operate the HHPA service and where individuals were receiving the usual available support services. All participants received a copy of the Pavement, a dedicated magazine for people who are homeless which lists relevant local services, and were reimbursed £10 cash for their time.^27^

### Baseline data collection

Peer advocates and co-researchers invited participants to complete a structured questionnaire (in English or Polish) on a tablet (Open Data Kit V.1.28.4) or administered in person, with 17 questionnaires conducted by phone. Questionnaire items were drawn from validated measures and other surveys^428^,^30^ and embedded in a linked qualitative study.^31^,^33^ Questions included demographic characteristics; indicators of multiple exclusion health; mental and physical health issues; health and social service use and barriers; and alcohol and other drug use.^4 28 30^ We measured symptoms of anxiety and depression using the Patient Health Questionnaire-4 (PHQ4),^34^ categorising participants with scores of 0–5 as having low-level symptoms, 6–8 as moderate and 9–12 as severe.^35^ (see online supplemental file 1 for the full questionnaire). Personal identifiers were used to link participants to three HES databases (England-wide) to extract attendance at scheduled outpatient appointments, use of A&E and inpatient admissions for 1 year prior to and post baseline interview.

### Outcomes

The primary outcome was not attending a scheduled outpatient appointment in a hospital (non-attendance) over 12 months post-recruitment, commencing from their baseline interview date. Secondary outcomes included the number of A&E services and inpatient admissions (all and planned admissions) during that same period.

### Confounders

Prespecified confounders included: age; gender (male, female and other); ethnicity (white only, black only and other); time since first episode of homelessness; citizenship (UK vs non-UK), all of which were self-reported, and history of prior hospital use from the linked HES data.^36^ Potential confounders included: historic indicators of exclusion (past injecting drug use, imprisonment and begging (asked passers-by for money in a public place)); current indicators of exclusion (location for sleeping last night categorised as stable (hostel, supported housing and own tenancy) and non-stable (rough, sofa surfing, friends, emergency, bed and breakfast accommodation); use of heroin in the last 12 months); reporting transportation as a barrier to healthcare; and symptoms of anxiety or depression.

### Missing data

Primary analyses assumed participants with at least one record in any of the six HES datasets in the 12 months pre-recruitment or post-recruitment had zero visits for their other datasets for which records were not found.

### Statistical analyses

We compared characteristics of clients and non-clients and linked and unlinked participants using medians and IQRs for continuous data and percentages for categorical data, and with standardised differences (for clients vs non-client comparisons).

We estimated the relative rate of non-attendance at an outpatient appointment using a weighted Poisson regression model among participants who had ≥1 appointments scheduled in the follow-up period. Models are offset for the number of appointments in that time. We used negative binomial models to estimate the predicted mean number of non-attended and attended outpatient appointments, A&E visits and inpatient admissions for clients and non-clients.

First, we present univariable associations for all outcomes. Second, potential confounders were included where there was a standardised difference >0.1 for linked clients compared with linked non-clients at baseline.^22^ To account for confounding, we calculated propensity scores in a logistic regression model where the outcome was whether the participant was a peer advocacy client. We combined similar categories with small strata (n<10) and considered interaction terms to better fit this model. The final propensity score model included two interactions: (1) ethnicity with historic count of non-attendance and (2) ethnicity and years since first became homeless. With the final propensity score model, we calculated the stabilised inverse probability of treatment weights and recalculated the standardised differences with these weights. We defined variables as persistently imbalanced where the weighted standardised difference was >0.1 (online supplemental file table S1). We weighted the regression analyses with the inverse probabilities.^22^ Third, we repeated the weighted regression analyses including the persistently imbalanced variables as covariates. For predicting means from the covariate-adjusted negative binomial regression models, we set the covariates to their mean values.

### Sensitivity and exploratory analyses

Sensitivity analyses assumed that anyone not linked in any of the databases did not have a scheduled outpatient appointment, or attend it, nor attend A&E, nor had an inpatient admission during the 12-month follow-up and were included in the analysis with 0 visits imputed. Exploratory analyses focused on repeating the weighted models by their baseline depression-anxiety symptom score, through the PHQ4 tool. We also examined differences between types of engagement with HHPA. This included examining differences between ‘new’ clients defined as those who had 0–1 engagements (any type) versus ‘ongoing’ clients defined as those ≥2 engagements scheduled with the peer advocacy programme at the point of study recruitment. To examine the reduced service that HHPA was conducting, we also examined differences between clients who had ≥2 peer-supported contacts consisting of a welfare visit, support with GP registration and accompaniment to a healthcare (hospital, GP and dentist) appointment (termed supported clients) compared with those who had either 0 or 1 contacts (termed unsupported) and mostly using the programme for assistance with travel during the study observation period.

## Results

A total of 311 participants were recruited, 153 HHPA clients (35.2%, n=434) and 158 non-clients (91.3%, n=164 approached). Overall, 78.5% were male, and the median age was 48 years. Compared with non-clients, clients were older (50 years, IQR 43–58 vs 45 years, IQR 39–55) and had a longer duration of homelessness (20 years, IQR 7–33 vs 9 years, IQR 3–17). Fewer clients had slept outside the previous night compared with non-clients (1.3% vs 13.9%), but they were more likely to have past experience of injecting drugs (40.5% vs 17.7%), begging (51.0% vs 31.6%) or incarceration (58.8% vs 41.1%) and using heroin in the last year (40.0% vs 19.0%) compared with non-clients. Across both groups, 38.2% had severe symptoms of depression or anxiety (table 1).

**Table 1:** Characteristics of all participants and by use of peer advocacy intervention

	HHPA clients (n=153)	Non-clients (n=158)	Total (n=311)
	n (col %)	n (col %)	n (col %)
Gender			
Male	118 (77.1)	126 (79.7)	244 (78.5)
Female	35 (22.8)	32 (20.2)	67 (21.5)
Non-binary, genderqueer/fluid, agender	<5	<5	<5
Age, years (median, IQR)	50 (43–58)	45 (39–55)	48 (40–57)
Sexual orientation			
Heterosexual	138 (92.0)	132 (83.5)	270 (86.8)
Gay, lesbian, bisexual, other	15 (9.9)	25 (15.8)	41 (13.2)
Ethnicity			
White only	106 (69.3)	97 (61.4)	203 (65.3)
Black/Black British only	12 (7.8)	15 (9.5)	27 (8.7)
Asian, Other, multiple, refuse	35 (22.9)	46 (29.1)	81 (26.0)
Citizenship			
Other	26 (17.0)	42 (26.5)	68 (21.9)
British	127 (83.0)	116 (73.4)	243 (78.1)
Education completed			
Less than secondary	18 (11.8)	22 (14.2)	40 (13.0)
Secondary	88 (57.9)	73 (47.1)	161 (52.4)
More than secondary	46 (30.3)	60 (38.7)	106 (34.5)
Duration homeless years (median, IQR)	20 (7–33)	9 (3–17)	11 (5–28)
Sleeping location, last night			
Slept rough/public location	2 (1.3)	22 (13.9)	24 (7.7)
Hostel	104 (68.0)	114 (72.1)	218 (70.1)
Own tenancy	27 (17.6)	5 (3.2)	32 (10.3)
Other	20 (13.1)	17 (10.8)	37 (11.9)
Past experience of multiple exclusion			
Begged	78 (51.0)	50 (31.6)	128 (41.2)
Sold sex	15 (9.8)	15 (9.5)	30 (9.6)
Incarcerated	90 (58.8)	65 (41.1)	155 (49.8)
Injected drugs	62 (40.5)	28 (17.7)	90 (28.4)
Healthcare barriers, current*			
Problems with transportation	105 (68.6)	83 (52.5)	188 (60.5)
Uncertainty about place and provider	96 (62.7)	89 (56.3)	185 (59.5)
Couldn’t get appointment	87 (56.9)	74 (46.8)	161 (51.8)
Used heroin in last 12 months			
No	92 (60.1)	128 (81.0)	220 (70.7)
Yes	61 (40.0)	30 (19.0)	91 (29.2)
PHQ4 score category			
0–5 (low)	55 (36.9)	57 (36.8)	112 (36.8)
6–8 (moderate)	37 (24.8)	39 (25.2)	76 (25.0)
9–12 (severe)	57 (38.3)	59 (38.1)	116 (38.2)

* Participants could select more than one option for each of these measures. Only the stratum with the affirmative response is presented here. Not all percentages will add up to 100% due to non-response or don’t know responses.

HHPA, Homeless Health Peer Advocate; PHQ4, 4 item Patient Health Questionnaire to measure symptoms of anxiety or depression.

### Characteristics of participants linked into HES

Overall, 229/311 participants (73.6%) were linked to at least one record in an HES dataset (outpatient, inpatient or A&E) in the 12 months either pre-enrolment or postenrolment. A total of 82 participants (26.4%) had no records located in any dataset and were unlinked. Among clients, 84.3% were linked to an HES record compared with 63.3% of non-clients. A total of 174 participants had ≥1 attendance at an outpatient appointment, 196 had used A&E services and 126 had an inpatient admission. Table 2 summarises the recruitment and data linkage of peer advocacy clients and non-clients. Subgroups less likely to link included those identifying as Asian/multiple/other ethnicity (34.2%), those with non-UK citizenship (30.5%), those with less than secondary school education (18.3%), and those reporting an unstable sleeping location last night (26.8%). The differences between linked peer advocacy clients and linked non-clients mirrored differences within the total sample (online supplemental table 1).

**Table 2:** Linkage of cohort study participants with Hospital Episode Statistics records for the 12 months prerecruitment and postrecruitment, London, UK

	HHPA clients	Non-clients
	n (%)	n (%)
Total recruited	153 (100)	158 (100)
Prerecruitment		
Outpatient	92/153 (60.5)	60/158 (38.0)
A&E	94/153 (61.4)	64/158 (40.5)
Inpatient	68/153 (44.4)	33/158 (20.9)
Postrecruitment		
Outpatient	114/153 (74.5)	60/158 (38.0)
A&E	95/153 (62.1)	55/158 (34.8)
Inpatient	68/153 (44.4)	21/158 (13.3)
Prerecruitment or postrecruitment		
Any appointment	129/153 (84.3)	100/158 (63.3)

A&E, accident and emergency; HHPA, Homeless Health Peer Advocacy.

### Hospital attendance among linked participants

For both clients and non-clients, the median number of scheduled and completed outpatient appointments was higher among participants at 12 months pre-recruitment compared with 12 months post-recruitment (table 3). The number of A&E visits was slightly higher among HHPA clients prerecruitment compared with postrecruitment but the same for non-clients. Between arms, there were large standardised differences across all the hospital outcome measures both before and after study recruitment. Non-clients had one scheduled outpatient appointment in the 12 months before recruitment, compared with four appointments for clients (standardised difference=0.50), and of these appointments, 0.0 ended in a non-attendance for non-clients compared with one for clients (standardised difference=0.45). After generating stabilised inverse probability of treatment weights, the only potential confounding variables where the weighted standardised differences remained >0.10 were the hospital use variables for the 12 months before study recruitment and years since first episode of homelessness; these were included in the weighted and adjusted models. Variables hypothesised as potential confounders which had weighted standardised differences <0.10 were not included in the adjusted models and are reported here.

**Table 3:** Hospital engagements in the last 12 months prerecruitment and postrecruitment for linked participants (n=229) and by intervention arm

Hospital engagement in 12 months	All	Non-clients	Clients	Standardised difference*	
	Median (IQR)/total (%)			Unweighted	Weighted
Prerecruitmentx					
Total outpatient appointments scheduled	1328	350	978		
Outpatient appointments scheduled	3.0 (0–8)	1.0 (0–5)	4 (0–10)	0.50	0.20
0	77 (33.6)	40 (40.0)	37 (28.7)		
1 or 2	35 (15.3)	22 (22.0)	13 (10.1)		
3+	117 (51.1)	38 (38.0)	79 (61.2)		
Outpatient appointments completed	4.0 (1–7)	2.5 (1–5)	4 (2–7.5)		
0	13 (8.6)	7 (11.7)	6 (6.52)		
1 or 2	50 (32.9)	23 (38.3)	27 (29.3)		
3+	89 (58.5)	30 (50.0)	59 (64.1)		
Total number of non-attendances†	268	70	198		
Outpatient non-attendances†	0 (0–1)	0 (0–1)	1 (0–2)	0.45	0.21
0	129 (56.3)	65 (65.0)	64 (49.6)		
1	43 (18.8)	18 (18.0)	25 (19.4)		
2+	57 (24.9)	17 (17.0)	40 (31.0)		
Emergency department visits	1.0 (0–4)	1.0 (0–3)	2.0 (0–4)	0.36	0.19
0	71 (31.0)	36 (36.0)	35 (27.1)		
1	86 (37.6)	37 (37.0)	49 (38.0)		
2+	72 (31.4)	27 (27.0)	45 (34.9)		
All inpatient admissions	0.0 (0–2)	0.0 (0–1)	1.0 (0–3)	0.45	0.23
0	128 (55.9)	67 (67.0)	61 (47.3)		
1	39 (17.0)	15 (15.0)	24 (18.6)		
2+	62 (27.1)	18 (18.0)	44 (34.1)		
Planned inpatient admissions	0.0 (0–0)	0.0 (0–0)	0.0 (0–0)		
0	192 (83.4)	89 (89.0)	103 (79.8)		
1	24 (10.5)	6 (6.0)	18 (14.0)		
2+	13 (5.7)	5 (5.0)	8 (6.2)		
Postrecruitment					
Total outpatient appointments scheduled	1251	500	751		
Outpatient appointments scheduled	4 (1–10)	2 (0–7)	6 (2–13)		
0	55 (24.0)	40 (40.0)	15 (11.6)		
1 or 2	34 (14.8)	15 (15.0)	19 (14.7)		
3+	140 (61.4)	45 (45.0)	95 (73.6)		
Outpatient appointments completed	3 (1–7)	3.0 (1–5.5)	3.0 (1–8)		
0	16 (9.2)	7 (11.7)	9 (7.9)		
1 or 2	58 (33.3)	18 (30.0)	40 (35.1)		
3+	100 (57.5)	35 (58.3)	65 (57.0)		
Total number of non-attendances†	395	112	283		
Outpatient non-attendances†	1 (0–2)	0 (0–2)	1 (0–3)		
0	103 (45.0)	57 (57.0)	46 (35.7)		
1 or 2	64 (27.9)	22 (22.0)	42 (32.5)		
3+	62 (27.1)	21 (21.0)	41 (31.8)		
Emergency department visits	1.0 (0–3.0)	1.0 (0–2)	2.0 (0–4)		
0	79 (34.5)	45 (45.0)	34 (26.4)		
1	80 (34.9)	32 (32.0)	8 (37.2)		
2+	70 (30.6)	23 (23.0)	47 (36.4)		
Inpatient admission	0.0 (0–2)	0.0 (0–0)	1.0 (0–3)		
0	140 (61.1)	79 (79.0)	61 (47.3)		
1	28 (12.2)	9 (9.0)	19 (14.7)		
2+	61 (26.6)	12 (12.0)	49 (38.0)		
Planned inpatient admissions	0.0 (0–0)	0.0 (0–0)	0.0 (0–0)		
0	191 (83.4)	92 (92.0)	99 (76.7)		
1	22 (9.6)	3 (3.0)	19 (14.7)		
2+	16 (7.0)	5 (5.0)	11 (8.5)		

* Standardised differences estimated for potential confounders to inform propensity score model.

† Among those with an outpatient appointment scheduled in the same time period.

### Effect of peer advocacy

In the weighted regression model (table 4), we found no evidence of a difference between clients and non-clients in the probability of non-attendance at an outpatient appointment in the 12 months post-recruitment (rate ratio (RR) 0.97 (95% CI 0.67 to 1.42)). The RR was similar in the weighted model adjusted for imbalanced confounders (RR 1.03 (95% CI 0.69 to 1.54)). We found no evidence of difference in the number of non-attendances at outpatient appointments, the number of completed outpatient appointments, the number of A&E visits or planned inpatient admissions between arms. In a weighted negative binomial model, we estimated that clients had an estimated mean of 1.65 for all types of inpatient admissions (95% CI 1.10 to 2.20) in the 12 months following recruitment, which was more than the 0.54 admissions for non-clients (95% CI 0.27 to 0.82). Similar results were observed in the weighted multivariable model.

**Table 4:** Effect of being a peer advocacy client on hospital use among Hospital Episode Statistics-linked cohort study participants who are homeless in London, UK, 2021–2022

Outcome		Unadjusted	Weighted*	Weighted and adjusted†
Model including linked participants only		Estimated rate ratio (95% CI)		
Non-attendance at outpatient appointment	HPPA vs non-client	1.01 (0.75 to 1.35)	0.97 (0.67 to 1.42)	1.03 (0.69 to 1.54)
Frequency of visits/non-attendance		Predicted number of events (95% CI)‡		
Non-attendances at outpatient appointments	HHPA client	2.48 (1.99 to 2.40)	2.31 (2.71 to 2.91)	2.22 (1.56 to 2.87)
	Non-client	1.87 (1.33to 2.41)	1.89 (1.39 to 2.37)	1.84 (1.35 to 2.32)
Completed outpatient appointments	HHPA client	6.37 (5.1to 7.65)	5.86 (4.43 to 7.29)	5.70 (2.77 to 6.21)
	Non-client	5.11 (3.68 to 6.55)	4.93 (2.71 to 7.16)	4.49 (2.78 to 6.20)
Completed A&E visits	HHPA client	3.16 (2.41 to 3.90)	2.59 (1.93 to 3.24)	2.05 (1.55 to 2.55)
	Non-client	1.93 (1.30 to 2.48)	1.76 (1.13 to 2.40)	1.62 (1.11 to 2.13)
All inpatient admissions	HHPA client	2.01 (1.48 to 2.64)	1.65 (1.10 to 2.20)	1.41 (0.96 to 1.87)
	Non-client	0.50 (0.28 to 0.71)	0.54 (0.27 to 0.82)	0.54 (0.27 to 0.81)
Planned inpatient admissions	HHPA client	0.58 (0.29 to 0.87)	0.44 (0.11 to 0.77)	0.29 (0.14 to 0.44)
	Non-client	0.14 (0.04 to 2.41)	0.14 (0.04 to 0.24)	0.15 (0.04 to 0.27)
Sensitivity analysis with all participants				
Non-attendances at outpatient appointments	HHPA client	1.85 (1.35 to 2.35)	1.57 (1.11 to 2.03)	1.32 (0.83 to 1.80)
	Non-client	0.71 (0.49 to 0.92)	0.89 (0.58 to 1.20)	0.79 (0.54 to 1.04)
Completed outpatient appointments	HHPA client	4.75 (3.35 to 6.15)	4.01 (2.86 to 5.15)	3.29 (2.16 to 4.43)
	Non-client	1.94 (1.36 to 2.53)	2.24 (1.08 to 3.40)	1.74 (1.14 to 2.34)
Completed A&E visits	HHPA client	2.66 (1.93 to 3.39)	2.04 (1.49 to 2.58)	1.44 (1.02 to 1.86)
	Non-client	1.22 (0.87 to 1.57)	1.23 (0.81 to 1.63)	1.02 (0.73 to 1.31)
All inpatient admissions	HHPA client	1.70 (1.12 to 2.27)	1.33 (0.85 to 1.81)	1.02 (0.64 to 1.41)
	Non-client	0.32 (0.18 to 0.45)	0.39 (0.17 to 0.61)	0.36 (0.16 to 0.55)
Planned inpatient admissions	HHPA client	0.49 (0.24 to 0.74)	0.33 (0.10 to 0.57)	0.20 (0.09 to 0.31)
	Non-client	0.09 (0.03 to 0.15)	0.08 (0.02 to 0.15)	0.09 (0.02 to 0.16)

* Weights account for education, gender, sexual identity, ethnicity, age, duration of homelessness, history of begging, injecting drug use, incarceration, location sleeping last night, transportation problems, PHQ4 symptoms, use of heroin in the past 12 months and number of outpatient appointments, number of non-attendances at outpatient appointments and number of A&E visits scheduled in 12 months before study recruitment.

† Adjusted for years since first homeless and hospital use variables for the 12 months before study recruitment (ie, number of scheduled outpatient visits, number of non-attendance at outpatient visits, number of emergency department visits and number of inpatient admissions).

‡ Predicted at the mean values for covariates in the weighted and adjusted model.

A&E, accident and emergency; HHPA, Homeless Health Peer Advocacy; PHQ4, Patient Health Questionnaire-4.

### Sensitivity and exploratory analyses

In sensitivity analyses using weighted negative binomial models, clients had higher estimated mean numbers of completed outpatient appointments (4.01 (95% CI 2.86 to 5.15) vs 2.24 (95% CI 1.08 to 3.40)), outpatient non-attendances (1.57 (95% CI 1.11 to 2.03) vs 0.89 (95% CI 0.58 to 1.20)) and completed A&E visits (2.04 (95% CI 1.49 to 2.58) vs 1.23 (95% CI 0.81 to 1.63)) than for non-clients, although CIs overlap. Clients had higher estimated mean numbers of all inpatient admissions (1.33 (95% CI 0.85 to 1.85)) and planned inpatient admissions (0.33 (95% CI 0.10 to 0.57)) (table 4).

There were 55 clients and 57 non-clients graded with low-level symptoms for anxiety or depression; 37 clients and 39 non-clients were categorised with moderate, and 57 clients and 59 non-clients with severe symptoms (table 1). Clients categorised with severe symptoms had 1.98 (95% CI 1.00 to 3.89) higher risk of non-attendance at an outpatient appointment compared with non-clients with severe symptoms. Among participants with moderate symptoms, there was some evidence that the adjusted mean number of completed outpatient appointments was higher for clients (5.86 (95% CI 2.73 to 9.00)) than for non-clients (1.87 (95% CI 0.41 to 3.34)). Clients with moderate symptoms of anxiety or depression also had a higher adjusted mean number of inpatient admissions (1.13 (95% CI 0.66 to 1.61) vs 0.13 (95% CI −0.01 to 0.27)) than for non-clients. Similarly, the adjusted mean number of planned inpatient admissions was higher among clients with moderate symptoms (0.47 (95% CI 0.10 to 0.85)) (table 5).

**Table 5:** Exploratory analyses: effect of peer advocacy on hospital use within categories of anxiety to and depression symptoms, London, UK (2021–2022)

Outcome		Low (PHQ4 0–5)	Moderate (PHQ4 6–9)	Severe (PHQ4 10–12)
	Estimated rate ratio (95% CI)			
Non-attendance at outpatient appointment	HPPA vs non-client	0.95 (0.45 to 2.00)	0.68 (0.45 to 1.04)	1.98 (1.00 to 3.89)
Frequency of visits/non-attendance	Mean number of events (95% CI)			
Non-attendances at outpatient appointments	HHPA	1.94 (1.17 to 2.39)	2.62 (1.45 to 3.79)	2.53 (1.34 to 3.72)
	Non-client	2.13 (1.34 to 2.92)	1.72 (0.25 to 3.18)	1.77 (1.15 to 2.39)
Completed outpatient appointments	HHPA	6.36 (4.16 to 8.56)	5.86 (2.73 to 9.0)	5.56 (3.30 to 7.83)
	Non-client	3.11 (1.44 to 4.78)	1.87 (0.41 to 3.34)	7.37 (3.15 to 11.61)
Completed A&E visits	HHPA	2.00 (1.08 to 2.92)	2.79 (1.49 to 4.10)	2.97 (1.78 to 4.15)
	Non-client	2.12 (0.82 to 3.42)	1.52 (0.26 to 2.81)	1.57 (0.88 to 2.37)
All inpatient admissions	HHPA	2.00 (0.81 to 3.20)	1.13 (0.66 to 1.61)	1.62 (0.86 to 2.38)
	Non-client	0.80 (0.16 to 1.44)	0.13 (−0.01 to 0.27)	0.71 (0.25 to 1.16)
Planned inpatient admissions	HHPA	0.69 (−0.10 to 0.85)	0.47 (0.10 to 0.85)	0.16 (0.02 to 0.30)
	Non-client	0.15 (−0.03 to 0.34)	0 (0 to 0)	0.24 (0.03 to 0.44)

A&E, accident and emergency; HHPA, Homeless Health Peer Advocacy; PHQ4, Patient Health Questionnaire-4.

Overall, 80 (52.3%) were new clients and 73 (47.7%) were ongoing clients. There were 86 (56.2%) unsupported and 67 (43.8%) supported clients (table 6). There were few differences across any of the outcomes with the exception of use of A&E and inpatient admissions. New peer advocacy clients had an adjusted mean of 1.23 inpatient admissions (95% CI 0.73 to 1.72), ongoing clients had a mean of 2.15 admissions (95% CI 1.10 to 3.20), compared with 0.60 (95% CI 0.28 to 0.93) among non-clients. Compared with non-clients who had an adjusted mean of 0.60 inpatient admissions (95% CI 0.28 to 0.93), non-supported clients had an adjusted mean of 1.51 inpatient admissions (95% CI 0.89 to 2.13), while supported peer advocacy clients had a mean of 1.81 (95% CI 0.87 to 2.75). Finally, supported clients had a higher adjusted mean number of A&E visits (2.89 (95% CI 1.93 to 3.86)) compared with non-clients (1.78 (95% CI 1.11 to 2.56)).

**Table 6:** Exploratory analyses: effect of being a peer advocacy client on hospital use within client subgroups, London, UK, 2021–2022

Outcome	Non-client	New client	Ongoing client	Non-supported client	Supported client
	158	80	73	86	67
	Estimated rate ratio (95% CI)				
Non-attendance at outpatient appointment	1.0	1.03 (0.69 to 1.5)	0.90 (0.58 to 1.39)	1.02 (0.68 to 1.52)	0.90 (0.58 to 1.39)
Frequency of visits/non-attendance	Mean number of events (95% CI)				
Non-attendances at outpatient appointments	1.89 (1.37 to 2.41)	2.78 (1.83 to 3.72)	1.89 (1.20 to 2.59)	2.61 (1.69 to 3.54)	1.96 (1.32 to 2.61)
Completed outpatient appointments	4.72 (2.52 to 6.92)	6.56 (4.35 to 8.74)	5.20 (3.42 to 6.95)	6.10 (4.0 to 8.17)	5.65 (3.87 to 7.44)
Completed A&E visits	1.78 (1.11 to 2.56)	2.14 (1.41 to 2.87)	3.10 (1.93 to 4.28)	2.35 (1.50 to 3.20)	2.89 (1.93 to 3.86)
All inpatient admissions	0.60 (0.28 to 0.93)	1.23 (0.73 to 1.72)	2.15 (1.10 to 3.20)	1.51 (0.89 to 2.13)	1.81 (0.87 to 2.75)
Planned inpatient admissions	0.14 (0.04 to 0.25)	0.28 (0.07 to 0.49)	0.66 (−0.05 to 1.37)	0.26 (0.07 to 0.45)	0.75 (−0.05 to 1.55)

A&E, accident and emergency.

## Discussion

This study aimed to measure the effectiveness of peer advocacy on hospital use among people who are homeless in London. Ultimately, the study was conducted in the context of disruptions to both HHPA and healthcare appointments due to COVID-19. Findings were mixed. We found no evidence that peer advocacy reduced outpatient non-attendances or A&E use. However, peer advocacy clients had more total inpatient admissions in the 12 months following recruitment (1.65) compared with non-clients (0.54), a pattern that was consistent in sensitivity analyses. There was also some evidence in sensitivity analyses that clients had more A&E visits and completed outpatient appointments, but also more non-attendances. We found that the effectiveness of peer advocacy is linked to symptoms of anxiety and depression scores, but not to types of peer engagement. Peer advocacy clients linked to HES had a longer duration of homelessness and higher rates of overall exclusion, including a history of injecting drugs, incarceration and begging, as well as more heroin use in the last year compared with non-clients.

Previous evidence about peer interventions from the UK is inconclusive. One trial showed peer educators did not increase uptake of tuberculosis screening,^18^ while another found a positive impact on engagement with clinical hepatitis services.^15^ Peer support in this trial consisted of specialist training on hepatitis C management and intensive support during and between appointments. This contrasts with the reduced service that HHPA provided due to COVID-19. The lack of reduction in non-attendance at outpatient appointments is disappointing, and as a result we could not establish if HHPA is cost-saving.^22^ However, the association with peer advocacy and increasing inpatient admissions suggests that health needs are being addressed, facilitated by HPPA.^37^ What remains unclear is whether the higher number of inpatient admissions reflects a negative effect, such as conditions not being addressed early enough, or a positive effect, with HHPA engagement occurring when individuals are already symptomatic. The latter would imply that HHPA is facilitating access to needed care. The absence of a difference in unplanned admissions supports the interpretation that HHPA may be facilitating timely treatment. Additionally, the greater number of completed outpatient appointments among clients, as indicated in our sensitivity analysis, suggests that HHPA may also be facilitating earlier access to care.

Although peers are primarily focused on supporting issues affecting access to healthcare, our qualitative process evaluation shows that HHPA adapts to clients’ needs.^23^ This highlights the complexities of quantifying its effects. Successful healthcare interactions may be linked to episodes of care and contingent on the ongoing presence of the peer, while permanent changes in healthcare use require long-term and open-ended support. Overcoming barriers to attend inflexible appointments among this highly marginalised population, even with peer support, is complex and time-consuming. Overall, 20% of appointments resulted in non-attendance prior to recruitment; this is almost three times higher than the national prevalence of 7.6%.^38^ Most strategies and research to reduce non-attendance focus on changing behaviours of the individual rather than the context of healthcare. Peer advocacy challenges this by attempting to change the context of the healthcare appointment.^38^ Future impact evaluations should measure longer-term changes for individuals, including self-efficacy or systemic changes in healthcare. Our qualitative work also highlighted the importance of day centre and hostel settings in shaping the work of HHPA. This occurs through several mechanisms, including making referrals to HHPA, ongoing staff support throughout their HHPA engagement and also as a core determinant of clients and residents’ physical and mental health. Hostel and day centre contexts vary widely depending on funding, staffing levels and burnout, issues worsened by the pandemic’s effect on the sector. Future work also needs to explore the culture of care and support in these settings, particularly in relation to stigma and the extent to which the presence of peers within a hostel, day centre or care facility may change the norms of staff and providers, reducing stigma and discrimination.

Our finding of effectiveness of peer advocacy among participants with moderate symptoms of anxiety or depression—but not severe symptoms—is in line with review evidence.^13^ Further investigation on the role of peer advocacy and mental health is needed, particularly considering the high levels of anxiety and depression reported,^4 39^ and to inform the growing use of psychologically-informed approaches in homeless services.^40^ We urge caution that peer support is not prioritised for its lower costs rather than its specific benefits, given significant funding cuts to mental health services.

### Strengths and limitations

The use of an outcome measure derived from hospital data is a key strength of this evaluation. Prior research had been conducted by our team demonstrating the feasibility of measuring hospital discharges among people who are homeless via data linkage to HES.^41 42^ A small-scale evaluation of the HHPA programme demonstrated the feasibility of data linkage, while focus groups with people experiencing homelessness indicated that such linkage was acceptable to this population.^20 26^ However, we could not assume that data from HES were missing at random, nor did we have any validated records indicating true non-engagement with hospitals among those assigned zero records for their unlinked datasets. This could create bias in our sensitivity analysis. As noted in the methods, our sample size deviated from the original protocol. In addition to this, sample sizes were smaller than anticipated due to fewer scheduled outpatient appointments in the follow-up period as a result of COVID-19, as well as fewer non-clients linking to HES. Our sample size calculations assumed that 80% of the sample would be linked to HES data. While overall 84.3% of HHPA clients were linked, only 63% of non-clients were linked to any HES record, and only 38% were linked to outpatient records. This reduced the study’s ability to accurately reject the null hypothesis of no effect. The change in our inclusion criteria for HHPA clients—from newly enrolled clients to those who had used the service in the last 6 months—may also have introduced further bias into the analysis. First, it may have increased differences between clients and non-clients. Second, it may have reduced the observed difference in the frequency of non-attendance among HHPA clients before and after enrolment. Defining a comparison population was difficult given the broad criteria for HHPA engagement and is reflected in the differences observed between clients and non-clients. We tried to adjust for imbalances, but there may be unmeasured confounders.

## Conclusion

While we did not find any effect of peer advocacy on the probability of non-attendance at outpatient appointments, nor on the number of A&E visits, clients had more inpatient admissions. Sensitivity analysis suggested clients have more completed outpatient appointments, hospital admissions and A&E visits. Results demonstrate the importance of peer advocacy to address healthcare needs of people who are homeless and that effectiveness is linked to severity of anxiety or depression experienced by clients, information that can be used to inform scale-up of the intervention. Any certainty with which we can interpret these findings is challenged by the disruption caused to both the peer advocacy work and health services by COVID-19.

## Supplementary material

10.1136/bmjopen-2025-107422 online supplemental file 1

10.1136/bmjopen-2025-107422 online supplemental file 2

## Data Availability

Data are available upon reasonable request.
