# Supplementary material for: Peer advocacy and access to hospital care for people who are homeless in London, UK, 2019–2023: a cohort study
Source: BMJ Open. 2026 Jul 16;16(7):e107422. doi: 10.1136/bmjopen-2025-107422 (PMC13384207; doi:10.1136/bmjopen-2025-107422)
Supplement: online supplemental file 1 [file bmjopen-16-7-s001.docx]

Questionnaire sections

[Part 1 – Field Work Admin 2](#_Toc70584942)

[Pre-eligibility sociodems 5](#_Toc70584943)

[Part 2 - Sociodemographic characteristics 11](#_Toc70584944)

[Homelessness characteristics 13](#_Toc70584945)

[Health difficulties 15](#_Toc70584946)

[Health-related Self-efficacy 16](#_Toc70584947)

[Health-related social capital / HHPA exposure 17](#_Toc70584948)

[Covid vaccine uptake 18](#_Toc70584949)

[Depression & anxiety 19](#_Toc70584950)

[Substance use 20](#_Toc70584951)

[Sex work 21](#_Toc70584952)

[Violence 22](#_Toc70584953)

[Policing 23](#_Toc70584954)

[Digital Literacy 24](#_Toc70584955)

[Part 3 - HES linkage data 24](#_Toc70584956)

[Part 4 – Postscript 25](#_Toc70584957)

[Part 5 - Post-questionnaire documentation 26](#_Toc70584958)

| **Section/Question** | **Label** | **Value** | **Codebook**  **variable** | **Source** |
| --- | --- | --- | --- | --- |
| Part 1 – Field Work Admin |  |  |  |  |
| Welcome to the HHPA Evaluation |  |  |  |  |
| What is your name? | Alex M | 1 | cor |  |
|  | Attie M | 2 |  |  |
|  | Adrian G | 3 |  |  |
|  | Karen G | 4 |  |  |
|  | Michael M | 5 |  |  |
|  | Esi M | 6 |  |  |
|  | John D | 7 |  |  |
|  | Keely T | 8 |  |  |
|  | Lucy P | 9 |  |  |
|  | Marcin T | 10 |  |  |
|  | Maya P | 11 |  |  |
|  | Spike H | 12 |  |  |
|  | Sujit R | 13 |  |  |
|  | Tracey S | 14 |  |  |
|  | Adeola P | 15 |  |  |
|  | Angelo R | 16 |  |  |
|  | Atif K | 17 |  |  |
|  | Jason W | 18 |  |  |
| How are you conducting the interview? | In person | 2 | mode |  |
|  | Remotely (phone, video chat) | 1 |  |  |
| [For remote] Before calling the recruit confirm that you have the following | Contact details for recruit |  | remote |  |
|  | Contact details for venue staff |  |  |  |
|  | Link to The Pavement website |  |  |  |
|  | Communication device (phone/internet+headset) |  |  |  |
|  | Cash advance for e-vouchers (or Paniz/Mani to post voucher) |  |  |  |
| [For in person] Before departing for the venue, confirm that you have the following: | Participant pack (info sheet, consent form, The Pavement, business card) |  | checklist |  |
|  | Face Mask |  |  |  |
|  | Hand sanitizer |  |  |  |
|  | Incentive (in envelope) or e-voucher codes |  |  |  |
|  | Contact details for host at venue |  |  |  |
|  | LSHTM badge |  |  |  |
|  | LSHTM introduction letter |  |  |  |
|  | Mobile phone |  |  |  |
| [For in person] Before departing for the venue, consider whether you – or anyone in your household - have any of the following: | A new, dry cough [🡪 prompt to cancel] |  | covidsx |  |
|  | High temperature [🡪 prompt to cancel] |  |  |  |
|  | Loss / change of sense of smell / taste [🡪 prompt to cancel] |  |  |  |
| [Cancel prompt]  If you feel breathless: Use NHS 111 online https://111.nhs.uk/covid-19/  If you are struggling to breathe: Call 999  Otherwise, order a Coronavirus test: <https://www.nhs.uk/conditions/coronavirus-covid-19/testing-and-tracing/get-a-test-to-check-if-you-have-coronavirus/>  and stay at home until you get your result  Call Paniz to re-schedule.  [🡪 skip to end] | |  |  |  |
|  |  |  |  |  |
|  |  |  |  |  |
| Do you plan to interview a Groundswell HHPA (peer advocacy) client? | No | 0 | arm |  |
|  | Yes | 1 |  |  |
| Recruit is based in what borough? | Brent | 1 | borough |  |
|  | Camden | 2 |  |  |
|  | City of London | 3 |  |  |
|  | Croydon | 4 |  |  |
|  | Ealing | 5 |  |  |
|  | Hammersmith & Fulham | 6 |  |  |
|  | Hackney | 7 |  |  |
|  | Haringey | 8 |  |  |
|  | Hillingdon | 9 |  |  |
|  | Islington | 10 |  |  |
|  | Kensington & Chelsea | 11 |  |  |
|  | Lambeth | 12 |  |  |
|  | Lewisham | 13 |  |  |
|  | Newham | 14 |  |  |
|  | Redbridge | 15 |  |  |
|  | Richmond | 16 |  |  |
|  | Southwark | 17 |  |  |
|  | Tower Hamlets | 18 |  |  |
|  | Waltham Forest | 19 |  |  |
|  | Wandsworth | 20 |  |  |
|  | Westminster | 21 |  |  |
| [Controls only] Location of participants |  |  | venue |  |
| [In person only] AT THE VENUE, confirm that you have done the following: | Identified a contact person (e.g. hostel staff) |  |  |  |
|  | Located the nearest fire exit |  |  |  |
|  | Located the venue's evacuation meeting point |  |  |  |
|  | Found a handwashing facility |  |  |  |
|  | Called/texted Paniz / Mani to confirm your arrival |  |  |  |
| Now you can speak to a recruit for the study |  |  |  |  |
| Is the recruit fluent in English? | No |  | english |  |
|  | Yes |  |  |  |
| Is the recruit fluent in Polish? | No |  | polish |  |
|  | Yes |  |  |  |
| [If English=No AND Polish=No] The participant is ineligible. Go back and revise answers if appropriate, or swipe left to end the interview. |  |  |  |  |
| Pre-eligibility sociodems |  |  |  |  |
| I’m going to ask you a few questions to see if you are eligible for this study. |  |  |  |  |
| What is your date of birth? | dd-mm-yyyy |  | dob |  |
| To confirm, you are ${age} years old | No [🡪 go back] |  | ageconf |  |
|  | Yes |  |  |  |
| What best describes your gender | Male | 0 | gender | <https://www.stonewall.org.uk/sites/default/files/do_ask_do_tell_guide_2016.pdf>  removed ‘self-describe’ option and added more terms with non-binary  Adapted National LGBT survey 2018  <https://assets.publishing.service.gov.uk/government/uploads/system/uploads/attachment_data/file/721704/LGBT-survey-research-report.pdf> |
|  | Female | 1 |  |  |
|  | Non-binary / genderqueer / agender / gender fluid | 4 |  |  |
|  | Prefer not to say | 99 |  |  |
|  | Other | 77 |  |  |
| What is your ethnic group? [Choose all that apply.] | White |  | ethnic | Homeless Health Needs Audit (HHNA) #9, adapted by allowing multiple choices. Added Hispanic/Latino |
|  | Asian / British-Asian |  |  |  |
|  | Black / Black British |  |  |  |
|  | Arab |  |  |  |
|  | Hispanic / Latino |  |  |  |
|  | Other |  |  |  |
| Are you a citizen of… | United Kingdom |  | citizen |  |
|  | European Union country |  |  |  |
|  | Another country |  |  |  |
| Where did you slept last night? | Sleeping rough on streets/parks |  | sleepnow | HHNA #4 |
|  | In a hostel or supported accommodation |  |  |  |
|  | Squatting |  |  |  |
|  | Sleeping on somebody’s sofa/floor |  |  |  |
|  | In emergency accommodation, e.g. night shelter, refuge |  |  |  |
|  | In B&B or other temporary accommodation |  |  |  |
|  | Housed – in own tenancy [🡪 ineligible] |  |  |  |
|  | Other [🡪 ineligible] |  |  |  |
| Without going into detail, do you have any ongoing health issues?  Prompt: including mental health, substance use, and physical health | No [🡪ineligible] |  | ongoing |  |
|  | Yes |  |  |  |
| How easy is it for you to make and attend health appointments?  Prompt: Would you say it’s easy, a bit challenging, or almost impossible? | Easy [🡪 ineligible] | 1 | easy |  |
|  | Challenging | 2 |  |  |
|  | (Almost) Impossible | 3 |  |  |
| [Is the recruit cognitively able to give informed consent?] | No [🡪 reschedule prompt] |  | cognitive |  |
|  | Unsure [🡪 Prompt for witness] |  |  |  |
|  | Yes |  |  |  |
| Prompt for witness: Before we continue can I ask you to find a key worker who can be a witness for the next section? | No [🡪 deferral prompt] |  | witness |  |
|  | Yes |  |  |  |
| Deferral prompt: Let’s schedule a time to speak when a key worker can observe the informed consent process, and we will be able to continue. | [🡪 end] |  |  |  |
| [If ineligible]  For this study, we are looking for people who are homeless and who are struggling to meet their health care needs. It seems that I can’t recruit you for this study. Thank you for making the time to speak to me today. |  |  |  |  |
| You are eligible for the study, let me tell you more about it. | |  |  |  |
| [Information sheet script]  I’m part of a research team which is interested in people who are homeless in London. We want to know if health care use differs for people who have met a peer advocate, compared to people who haven’t. In London, peer advocates are trained and supported by Groundswell, a third sector organisation based in South London.  The research team includes people from the London School of Hygiene and Tropical Medicine, King’s College London and University College London. Findings from this research will be useful for local commissioners in London, as they make decisions about what services to offer for people who are homeless.  Study participants complete a questionnaire. The questionnaire takes about 25 minutes to complete, and has questions about your health status, drug and alcohol use, and health care use. You can refuse to answer any question I ask you.  I will need your permission for the research team to access your NHS records for the past and future 12 months, to find out more about how often you use outpatient care, how often you use A&E, and how often you are admitted for inpatient care. To access your NHS records, I will ask for your name and date of birth, and, if possible, your NHS number.  The research team is also interested in services offered for people who sleep rough. If you give us permission, we want to see if you have any records stored in the CHAIN database. It’s up to you whether you give us this permission.  Your data will be handled in confidence, stored on secure servers, and handled by a small number of researchers. We will store information about your name and date of birth separately from your responses to the rest of the questionnaire. We will not report your individual data to anyone. All our reports will be on a group level.  There is one exception to confidentiality, and that is if you say something which makes me think you are going to harm yourself or another person. In that case, I’ll stop the interview and we’ll talk about how to get you help from a key worker.  You can decline to participate in study. And if you do participate, you have the right to ask us to delete your data afterwards. This is possible up to the point of data analysis. I will give you our contact details when we’re done with the questionnaire.  Do you have any questions? I will be happy to clarify anything I’ve said. | |  |  | Information sheet |
| [If witness required] Name of witness |  |  | witnessname |  |
| I will read out a set of statements about this study. You need to agree with all the statements to be in the study. Say “yes” if you agree with the statement, or you can ask for clarification, or you can decline to participate in the study. | I understand the purpose of the study, what the study involves, and I understand why you would like to talk to me. |  | consent_ | Consent form |
|  | I have had the opportunity to ask any questions that I might have and am happy with the answers I have received. |  |  |  |
|  | I agree to complete the questionnaire. |  |  |  |
|  | I understand that I do not have to answer any question I do not want to, and that I can stop the interview at any time without giving a reason. |  |  |  |
|  | I understand that the information I give will be used in analysis and stored securely. |  |  |  |
|  | I understand that everything I say is confidential: researchers will not use my name when they write about this project. |  |  |  |
|  | I consent to giving my full name, date of birth, GP or home address and NHS number |  |  |  |
|  | I give permission for the team to obtain information from my health-related records and registers including from the National Health Service (NHS) (NHS registration, health status, treatment and use of health services, GPs, other healthcare organisations); as well as via NHS Digital, NHS Central Register, NHS Personal Demographics* Service and the Department of Health and Social Care* (*or successor organisations if these change) |  |  |  |
|  | I understand that any personal information that I give will be kept separately from my questionnaire data and destroyed at the end of the project. |  |  |  |
|  | I understand that if I tell you something that makes you think I or another adult is in immediate danger, or that a child is being harmed, you may have to tell someone what I have said. |  |  |  |
|  | I understand that my participation in the study is voluntary and that I am free to withdraw from the study at any time, without giving a reason. |  |  |  |
|  | I understand that I can request that my data be removed from the study up to the point of analysis, but not withdrawn afterwards. |  |  |  |
|  | I agree to take part in this study. |  |  |  |
|  | OPTIONAL: I give permission for the team to obtain information about my accommodation status and alcohol, drugs or mental health support needs, as well as my use of services relating to these needs, from the CHAIN database, if I have been recorded on it. |  |  |  |
| [If decline any consent items 1-13] Thank you for answering these questions. Only people who agree with all of the statements can participate in the study. Is there anything I can clarify about the study? If not, I want to thank you for taking the time to speak with me.  [🡪skip to end] |  |  |  |  |
| Part 2 - Sociodemographic characteristics |  |  |  |  |
| Thank you for agreeing to take part in this study.  Your answers to the following questions will be used to help us learn more about the health status of homeless people in London.  The only people who will see these answers will be members of the research team. | | | |  |
| You indicated an ethnic background which is white.  Which of these best describes your background? | British / English / Scottish / Welsh / No. Irish |  | ethwh |  |
|  | Irish |  |  |  |
|  | Gypsy or Irish Traveller |  |  |  |
|  | Other White |  |  |  |
| You indicated an ethnic background which is Asian.  Which of these best describes your background? | Bangladeshi |  | ethas |  |
|  | Chinese |  |  |  |
|  | Indian |  |  |  |
|  | Pakistani |  |  |  |
|  | Other Asian |  |  |  |
| You indicated an ethnic background which is African, Caribbean or Black British.  Which of these best describes your background? | African |  | ethbl |  |
|  | Caribbean |  |  |  |
|  | Other Black |  |  |  |
| Which of the following options best describes how you think of yourself? | Heterosexual / straight | 1 | sexorient | Adapted from Office for National Statistics (ONS) Annual Population Survey |
|  | Gay / lesbian | 2 |  |  |
|  | Bi | 3 |  |  |
|  | Don’t know | 88 |  |  |
|  | Refuse | 99 |  |  |
|  | Other | 77 |  |  |
| Do you identify as trans? | No | 0 | trans | Stonewall ‘Do ask, do tell’ <https://www.stonewall.org.uk/sites/default/files/do_ask_do_tell_guide_2016.pdf> |
|  | Yes | 1 |  |  |
|  | Refuse | 2 |  |  |
| How good are you at reading English when you need to in daily life? For example: reading newspapers and magazines or instructions for medicine or recipes? | Very good | 1 | reading | 2011 Skills for Life Survey, UK Dept for Business Innovation and skills  Bqread, |
|  | Fairly good | 2 |  |  |
|  | Below average | 3 |  |  |
|  | Poor | 4 |  |  |
|  | Cannot read English | 5 |  |  |
|  | Refuse | 99 |  |  |
| How good are you at writing in English when you need to in daily life? For example: writing letters or notes or filling in official forms? | Very good | 1 | writing | 2011 Skills for Life Survey, UK Dept for Business Innovation and skills  Bqwrite |
|  | Fairly good | 2 |  |  |
|  | Below average | 3 |  |  |
|  | Poor | 4 |  |  |
|  | Cannot write English | 5 |  |  |
|  | Refuse | 99 |  |  |
| Which of these categories best describes you at present? | Going to school or college full-time | 1 | employ | HHNA #2, added ‘unemployed and looking’ and ‘retired/ pensioner’ |
|  | In paid employment or self-employment | 2 |  |  |
|  | On a government scheme for employment training | 3 |  |  |
|  | Doing unpaid or voluntary work | 4 |  |  |
|  | Waiting to take up paid work already obtained | 5 |  |  |
|  | Intending to look for work but prevented by temporary sickness or injury | 6 |  |  |
|  | Permanently unable to work because of long-term sickness or disability | 7 |  |  |
|  | Unemployed and looking for work | 8 |  |  |
|  | Unemployed and not looking for work | 9 |  |  |
|  | Retired / pensioner |  |  |  |
|  | Other | 77 |  |  |
| Are you at present receiving any state benefits where you are the named recipient? | No | 0 | benefits_named | East London Project (ELP) Q13.05 |
|  | Yes [🡪] | 1 |  |  |
|  | Refuse | 99 |  |  |
| Are you eligible to receive state benefits? | No | 0 | benefits_elig |  |
|  | Yes | 1 |  |  |
|  | Refuse | 99 |  |  |
| Have you ever been refused housing or state benefits?? | No | 0 | benefits_refused | ELP Q13.05.01 |
|  | Yes | 1 |  |  |
|  | Refuse | 99 |  |  |
| What is the highest level of education that you have completed? | Further education beyond secondary/high school | 3 | edu |  |
|  | Secondary/high school | 2 |  |  |
|  | Primary | 1 |  |  |
|  | Less than primary | 0 |  |  |
| How many years have you lived in the United Kingdom? | __ years |  | inukyears |  |
| How old were you when you first became homeless? | …. years old |  | first |  |
| Homelessness characteristics |  |  |  |  |
| In your life, have you ever… | Stayed with friends, relatives or other people because you had no home of your own |  | meh | Multiple Exclusion Homeless (MEH) #1, from <https://doi.org/10.1017/S147474641100025X> |
|  | Stayed in a hostel, foyer, refuge, night shelter or B&B hotel because you had no home of your own |  |  | MEH #2 |
|  | Slept rough |  |  | MEH #3 |
|  | Applied to the council as homeless or as threatened with homelessness? |  |  | MEH #4 + input from Suzanne Fitzpatrick |
|  | Spent time in local authority care as a child |  |  | MEH #5 |
|  | Begged (that is, asked passers-by for money in the street or another public place) |  |  | MEH#12 |
|  | Shoplifted because you needed things like food, drugs, alcohol or money for somewhere to stay |  |  | MEH#14 |
| In the last 12 months, were you ever hungry and didn’t eat because you couldn’t afford enough food? | No | 0 | food | CDC NHANES |
|  | Yes | 1 |  |  |
|  | Don’t know | 88 |  |  |
|  | Refuse | 99 |  |  |
| Are you a caregiver for anyone? | No [🡪next section] | 0 | caregiver | Adapted from ELP Q13.02 |
|  | Yes | 1 |  |  |
| Who do you care for?  [Choose all that apply] | Children or dependents under 18 years |  | carefor |  |
|  | Children or dependents over 18 years |  |  |  |
|  | Parents or other adult family members |  |  |  |
|  | Friend(s) |  |  |  |
|  | Other |  |  |  |
| Health difficulties |  |  |  |  |
| The next few questions are about your health status. | | | |  |
| Do you currently have any of the following health problems?  Chose all that apply. | Asthma |  | health_  _asthma | Diseases from HHNA 13 & 16, combined some conditions, dropped others. Added autism, dyslexia, brain injury |
|  | Autism |  | _autism |  |
|  | Brain injury |  | _brain |  |
|  | Cancer |  | _cancer |  |
|  | Chronic breathing problems (bronchitis, emphysema, obstructive airways disease) |  | _breathe |  |
|  | Depression or anxiety |  | _depress |  |
|  | Diabetes |  | _diabetes |  |
|  | Difficulty seeing / eye problems |  | _vision |  |
|  | Drug (addiction) problems |  | _drug |  |
|  | Dyslexia |  | _dyslexia |  |
|  | Epilepsy / seizures |  | _epilepsy |  |
|  | Foot problems |  | _foot |  |
|  | Heart problems (including heart attack, angina, murmur, abnormal rhythm) |  | _heart |  |
|  | Hepatitis C |  | _hepc |  |
|  | High blood pressure |  | _hyperten |  |
|  | HIV |  | _hiv |  |
|  | Joint, bone or muscle problems |  | _jointbone |  |
|  | Psychosis / bipolar disorder |  | _psychosis |  |
|  | Sexually transmitted infection (chlamydia, gonorrhoea or pelvic inflammatory disease) |  | _sti |  |
|  | Skin/wound infection |  | _skin |  |
|  | Teeth / dental problems |  | _dental |  |
|  | Tuberculosis |  |  |  |
|  | Other health problems |  | _other |  |
| The next questions are about difficulties that people have when they are managing their health. | | | |  |
| Have you faced any of these difficulties when managing your health? Chose all that apply. | I was concerned about how much money it would cost. |  | barriers_ | Composite International Diagnostic Interview (CIDI) Services module, SR116 / SR 126  Cut first several items e.g. ‘insurance’.  ‘Transportation’ is simplified. |
|  | I was concerned about what people would think if they found out I was seeking treatment |  |  |  |
|  | I had problems with transportation |  |  |  |
|  | I was unsure about where to go or who to see |  |  |  |
|  | I thought it thought it would take too much time or be inconvenient |  |  |  |
|  | I could not get an appointment |  |  |  |
|  | I was scared about being put in a hospital against my will |  |  |  |
|  | I was dissatisfied with services I received in the past |  |  |  |
|  | I had experience with health workers who had not listened to my concerns |  |  |  |
|  | I had experience with healthcare workers who thought that I’m milking the system, e.g. trying to con them into giving me prescription medications to get high or sell. |  |  | from [10.1016/ j.drugalcdep.2016.02.019](https://dx.doi.org/10.1016%2Fj.drugalcdep.2016.02.019), Items 4,5,6  “pill shopping” 🡪 “milking the system” |
| [Of the options selected in health_***] Which of these health problems do you have **most** difficulty managing?  [Choose one] |  |  |  |  |
| Health-related Self-efficacy | | | | Groundswell HHPA Planning and Debriefing Tool.  Simplified answers to the medication item. |
| You have said that X is your most challenging health problem to manage. |  |  |  |  |
| Do you feel you understand the X and what might have caused it? | Yes – very clear on it | 2 | pdt_understand |  |
|  | I have an OK grasp on it but have some questions | 1 |  |  |
|  | I don’t know – need to ask in the next appointment | 0 |  |  |
| Do you feel confident to talk to the medical staff about X? | Yes, totally confident | 2 | pdt_confident |  |
|  | Mostly confident | 1 |  |  |
|  | No – not very confident, I need help to explain to the doctor | 0 |  |  |
| Do you know the different treatment options or medications that are available for X? | Yes – very clear on it | 2 | pdt_options |  |
|  | I have an OK grasp on it but have some questions | 1 |  |  |
|  | I don’t know – need to ask in the next appointment | 0 |  |  |
| Are you able to manage use any medication that you have been prescribed for X on an ongoing basis? | Yes | 1 | pdt_manage |  |
|  |  |  |  |  |
|  | No | 0 |  |  |
|  | Not applicable (no medications) | 66 |  |  |
| Health-related social capital / HHPA exposure | | | |  |
| In the past year, has anyone helped you out when you had a medical appointment? | No [🡪next section] | 0 | helped |  |
|  | Yes | 1 |  |  |
|  | Don’t know / don’t remember [🡪next section] | 88 |  |  |
| Did any of the following people help you?  [chose all that apply] | Family |  | helper |  |
|  | Friends |  |  |  |
|  | Neighbours |  |  |  |
|  | Hostel staff |  |  |  |
|  | Day centre staff |  |  |  |
|  | Volunteers or Charity workers, including outreach workers |  |  |  |
|  | Religious leaders |  |  |  |
| Did any of the people who helped you have lived experience with homelessness? | No [🡪] | 0 | livedexp |  |
|  | Yes | 1 |  |  |
|  | Don’t know | 88 |  |  |
| Were any of the people who helped you from Groundswell? | No | 0 | groundswell |  |
|  | Yes | 1 |  |  |
|  | Don’t know | 88 |  |  |
| In the past 12 months have you:  [Chose all that apply] | Seen a GP |  | services | HHNA #27, turned into binary responses, removed ‘homeless healthcare service’, added ‘dentist’ and OST |
|  | Been to A&E |  |  |  |
|  | Used an ambulance |  |  |  |
|  | Been admitted to hospital |  |  |  |
|  | Been to a dentist |  |  |  |
|  | Visited or been visited by someone working for a sex worker project? |  |  |  |
|  | Used drug or alcohol services? |  |  |  |
|  | Used substitute drug regime (e.g. methadone script) |  |  |  |
|  | Been visited by someone from Find and Treat |  |  |  |
|  | Been helped by a care navigator at Pathways |  |  |  |
| Covid vaccine uptake |  |  |  |  |
| Has anyone offered you a Covid vaccine? | No | 0 | covaxoffer |  |
|  | Yes | 1 |  |  |
|  | Don’t know | 88 |  |  |
|  | Refuse | 99 |  |  |
| Who offered you the Covid vaccine? | Invited by GP to receive a vaccine at a health centre / hospital / pharmacy |  | covaxprovider |  |
|  | Approached by roving (mobile) team |  |  |  |
|  | Other |  |  |  |
|  | Don't know / don't remember |  |  |  |
| Did you accept the vaccine? | No | 0 | covaxcaccept |  |
|  | Yes | 1 |  |  |
|  | Refuse | 99 |  |  |
| Depression & anxiety | | | | These are the 4 Qs used in ELP and Samvedana, day ranges adapted from Samvedana |
| The next four questions are about your feelings over the past two weeks. | | | |  |
| Over the past two weeks, how often have you been bothered by having Little interest or pleasure in doing things | Not at all (0 days) | 0 | phq1 | PHQ9 #1 |
|  | Several days (1 to 7 days) | 1 |  |  |
|  | More than half the days (8-11 days) | 2 |  |  |
|  | Nearly every day (12-14 days) | 3 |  |  |
| Over the past two weeks, how often have you been Feeling down, depressed, or hopeless | Not at all (0 days) | 0 | phq2 | PHQ9 #2 |
|  | Several days (1 to 7 days) | 1 |  |  |
|  | More than half the days (8-11 days) | 2 |  |  |
|  | Nearly every day (12-14 days) | 3 |  |  |
| Over the past two weeks, how often have you been Feeling nervous, anxious or on edge | Not at all (0 days) | 0 | phq3 | GAD7 #1 |
|  | Several days (1 to 7 days) | 1 |  |  |
|  | More than half the days (8-11 days) | 2 |  |  |
|  | Nearly every day (12-14 days) | 3 |  |  |
| Over the past two weeks, how often have you been Not able to stop worrying | Not at all (0 days) | 0 | phq4 | GAD7 #2 |
|  | Several days (1 to 7 days) | 1 |  |  |
|  | More than half the days (8-11 days) | 2 |  |  |
|  | Nearly every day (12-14 days) | 3 |  |  |
| In your life, have you ever been admitted to hospital with a mental health issue?  [This could be voluntarily or by being sectioned] | No [🡪] |  | admitted | MEH#7 |
|  | Yes |  |  |  |
|  | Refuse [🡪] |  |  |  |
| Has this happened in the last 6 months? | No |  | admitted6 |  |
|  | Yes |  |  |  |
|  | Refuse |  |  |  |
| Substance use |  |  |  |  |
| The next questions are about alcohol and drug use. Remember that your answers are confidential and are only used by members of the research team. | | | | ELP section Q12 |
| Have you ever had a period in your life when you had six or more alcoholic drinks on a daily basis? | No |  | meh_binge | MEH#11 |
|  | Yes |  |  |  |
|  | Refuse |  |  |  |
| In your life, have you ever been involved in street drinking?  By street drinking we mean heavy and/or frequent drinking in outdoor public places such as street, parks and public squares. | No |  | meh_streetdrink | MEH#13, prompt from Ross et at (2005) doi:10.1093/her/cyg118 and [here](http://www.wdp.org.uk/sites/default/files/content_pages/LBBD%20Street%20Drinking%20Report%202018%20%20FINAL.PDF) |
|  | Yes |  |  |  |
|  | Refuse |  |  |  |
| How often have you had an alcoholic drink during the past 12 months? | Seven days a week |  | drinkfreq | HHNA #22, CSEW (ALCOFT) |
|  | Five or six days a week |  |  |  |
|  | Three or four days a week |  |  |  |
|  | Once or twice a week |  |  |  |
|  | Once or twice a month |  |  |  |
|  | Once every couple of months |  |  |  |
|  | Once or twice a year |  |  |  |
|  | Not at all in the last 12 months |  |  |  |
|  | Refuse |  |  |  |
| In your life, have you ever used drugs not for medical purposes such as heroin, crack, weed? | No [🡪] |  | meh_drug | MEH #8 |
|  | Yes |  |  |  |
|  | Refuse [🡪] |  |  |  |
| In your life, have you ever injected drugs not for medical purposes such as heroin or crack? | No [🡪] |  | meh_inject | MEH #9 |
|  | Yes |  |  |  |
|  | Refuse [🡪] |  |  |  |
| [If ever inject is YES] In the past 12 months, have you injected any drugs? | No [🡪] |  | inject12 | Adapted from ELP Q12.06.01 |
|  | Yes |  |  |  |
|  | Refuse [🡪] |  |  |  |
| In the past 12 months, have you used needles/syringes that had been previously used by someone else? | No [🡪] |  | injectshare | ELP Q12.06.03 |
|  | Yes |  |  |  |
|  | Refuse [🡪] |  |  |  |
| Have you taken any of these drugs in the past year?  [Choose all that apply] | Did not take drugs in past 12 months |  | drug | HHNA 19 + ELP Q12.05.01 but with 12 month recall.  Removed the combo drugs, ecstasy, lsd, opium, ketamine, poppers, barbituates/downers, steroids, methadone, legal highs, marijuana  Added synthetic cannaboids (spice,) |
|  | Heroin | 1 |  |  |
|  | Crack | 2 |  |  |
|  | Powder cocaine (coke) | 3 |  |  |
|  | Fentanyl | 4 |  |  |
|  | Marijuana / Cannabis / Weed | 5 |  |  |
|  | Synthetic cannabinoids such as Spice (or black mamba, noids, clockwork orange) | 6 |  |  |
|  | Tranquilisers such as benzodiazepines/benzos | 7 |  |  |
|  | Crystal Methamphetamine | 8 |  |  |
| In the past 12 months, have you overdosed to the point where you lost consciousness? | No [🡪] | 0 | overdose | WHO drug use surveys |
|  | Yes | 1 |  |  |
|  | Refuse [🡪] | 99 |  |  |
| [If uses heroin] How often do you use heroin? | Nearly every day | 1 | dailyheroin |  |
|  | Less often | 0 |  |  |
| [If uses crack/cocaine] How often do you use crack / cocaine? | Nearly every day | 1 | dailycrack |  |
|  | Less often | 0 |  |  |
| [If uses spice] How often do you use spice? | Nearly every day | 1 | dailyspice |  |
|  | Less often | 0 |  |  |
| [If uses weed] How often do you use marijuana / cannabis / weed | Nearly every day | 1 | dailyweed |  |
|  | Less often | 0 |  |  |
| Sex work |  |  |  |  |
| In their lives, many people who are homeless find it difficult to engage in formal work and use different means to make money and go about their everyday life. | | | |  |
| Have you sold sex in the past 6 months? By selling sex, we mean exchanging sex for money, drugs or goods. | No | 0 | sexwork | ELP Eligibility Screener, with recall period of ever. |
|  | Yes | 1 |  |  |
|  | Refuse [🡪] | 99 |  |  |
| Have you ever sold sex? By selling sex, we mean exchanging sex for money, drugs or goods. | No | 0 | sexwork6 | ELP Eligibility Screener, with recall period of 6 mo. |
|  | Yes | 1 |  |  |
|  | Refuse | 99 |  |  |
| Violence |  |  |  |  |
| In their lives, many people who are homeless experience different forms of violence from relatives, people that they know and/or from strangers. I would like to ask you about some of these situations. This is important to know in order to understand all the issues people who are homeless experience that might affect their health and well-being. You do not have to answer any of these questions, but remember that if you do, all your answers are confidential. | | | | Preface adapted from WHO Multi-country study on Women’s Health and Life Events Version 9.9.  Questions adapted from ELP Section 5. Change from ‘a client’ to ‘another person’ |
| In the past 6 months, has another person verbally, physically or sexually abused you? | No [🡪] | 0 | viosix |  |
|  | Yes | 1 |  |  |
|  | Don’t know [🡪] | 88 |  |  |
|  | Refuse [🡪] | 99 |  |  |
| Did you experience verbal abuse?  Prompt: that is to say they belitted or humiliated you or used abusive or insulting language towards you such as calling you inappropriate names or making racist remark? | No [🡪] | 0 | viosix_verbal |  |
|  | Yes | 1 |  |  |
|  | Don’t know [🡪] | 88 |  |  |
|  | Refuse [🡪] | 99 |  |  |
| Did you experience physical abuse?  [Prompt] That is to say you were pushed, shoved, slapped, kicked, punched, choked, dragged, burned you, or used a weapon against you, thrown something at you, or beaten you up? | No[🡪] | 0 | viosix_physical |  |
|  | Yes | 1 |  |  |
|  | Don’t know[🡪] | 88 |  |  |
|  | Refuse[🡪] | 99 |  |  |
| Did you experience sexual abuse?  [Prompt] That is to say you were touched or grabbed you sexually against your will (grope) or attempted to get sex through force. | No[🡪] | 0 | viosix_sexual |  |
|  | Yes | 1 |  |  |
|  | Don’t know[🡪] | 88 |  |  |
|  | Refuse[🡪] | 99 |  |  |
| Has another person EVER verbally, physically or sexually abused you? | No [🡪] | 0 | vioev |  |
|  | Yes | 1 |  |  |
|  | Don’t know [🡪] | 88 |  |  |
|  | Refuse [🡪] | 99 |  |  |
| Did you experience verbal abuse?  Prompt: that is to say they belitted or humiliated you or used abusive or insulting language towards you such as calling you inappropriate names or making racist remark? | No [🡪] | 0 | vioev_verbal |  |
|  | Yes | 1 |  |  |
|  | Don’t know [🡪] | 88 |  |  |
|  | Refuse [🡪] | 99 |  |  |
| Did you experience physical abuse?  [Prompt] That is to say you were pushed, shoved, slapped, kicked, punched, choked, dragged, burned you, or used a weapon against you, thrown something at you, or beaten you up? | No[🡪] | 0 | vioev_physical |  |
|  | Yes | 1 |  |  |
|  | Don’t know[🡪] | 88 |  |  |
|  | Refuse[🡪] | 99 |  |  |
| Did you experience sexual abuse?  [Prompt] That is to say you were touched or grabbed you sexually against your will (grope) or attempted to get sex through force. | No[🡪] | 0 | vioev_sexual |  |
|  | Yes | 1 |  |  |
|  | Don’t know[🡪] | 88 |  |  |
|  | Refuse[🡪] | 99 |  |  |
| Policing | | | | ELP Section 6 |
| People who are homeless are often more vulnerable to being approached by the police or security guards and this can have negative affects on other aspects of their lives. We wanted to ask you about your contact with the police or criminal justice service. We won’t ask you about the reasons for this contact, only if it happened. Remember that you don’t have to answer any question, but if you do it will remain confidential. | | | |  |
| In the last six months, have you been arrested or detained or charged by police in the UK (for any reason)? | No | 0 | arrestsix |  |
|  | Yes | 1 |  |  |
|  | Refuse | 99 |  |  |
| Have you EVER been arrested or detained or charged by police in the UK (for any reason)? | No [🡪 ] | 0 | arrestev |  |
|  | Yes | 1 |  |  |
|  | Refuse [🡪] | 99 |  |  |
| In the last six months, have you spent time in prison or a young offenders institute? | No [🡪 ] | 0 | prisonsix | ELP Section 6 |
|  | Yes | 1 |  |  |
|  | Refuse [🡪] | 99 |  |  |
| Have you EVER spent time in prison or a young offenders institute? | No [🡪 ] | 0 | prisonev | MEH #6 |
|  | Yes | 1 |  |  |
|  | Refuse [🡪] | 99 |  |  |
| In the last six months, has a police officer (including community police officers) or security guards asked you to move on from a public space? | No [🡪 ] | 0 | movesix |  |
|  | Yes | 1 |  |  |
|  | Refuse [🡪] | 99 |  |  |
| Has a police officer (including community police officers) or security guards EVER asked you to move on from a public space? | No [🡪 ] | 0 | moveev |  |
|  | Yes | 1 |  |  |
|  | Refuse [🡪] | 99 |  |  |
| Digital Literacy |  |  |  |  |
| These next questions are about use of mobile phones, this information will be used to develop services for people who are homeless in London. | | | |  |
| Do you own a mobile phone? | No [🡪next section] | 0 | mobile_own |  |
|  | Yes | 1 |  |  |
| Do you use this mobile to access the internet | No [🡪] | 0 | mobile_net |  |
|  | Yes | 1 |  |  |
| Do you use this mobile to manage your health care? | No [🡪] | 0 | mobile_use |  |
|  | Yes | 1 |  |  |
| Would you be willing to use your mobile to manage your health care? | No [🡪] | 0 | mobile_willing |  |
|  | Yes | 1 |  |  |
|  | | | |  |
| Part 3 - HES linkage data |  |  |  |  |
| The last few questions are so that the research team can locate your health records. Just to confirm, your answers are confidential, and are only used by people on the research team. | | | |  |
| What is your last name? |  |  | lname |  |
| What is your first name? |  |  | fname |  |
| What is your middle name? |  |  | mname |  |
| Do medical providers know you by any other name(s)? | No | 0 | anyalias |  |
|  | Yes | 1 |  |  |
| What other names are you known by? [Separate names with a comma e.g. “John, Jonathan, Johnny”] |  |  | aliases |  |
| Do you know your NHS number? [do you have access to your NHS number at this facility? If you are able to get the letter, or from the staff here, I’ll get your incentive ready while you get the letter/number] | No [🡪] | 0 | nhs |  |
|  | Yes | 1 |  |  |
| What is your NHS number? |  |  | nhsnumber |  |
| Do you have more NHS numbers? | No [🡪] | 0 | nhsmore |  |
|  | Yes | 1 |  |  |
|  | Refuse [🡪] | 99 |  |  |
| What are the NHS numbers? Separate each number with a comma. |  |  | nhsnumbers |  |
| Are you registered at a GP surgery? | No [🡪next section] | 0 | gpregister |  |
|  | Yes | 1 |  |  |
| What is the name of the surgery where you are registered? |  |  | gpname |  |
| Do you know the location of the surgery? | No [🡪] | 0 | gplocation |  |
|  | Yes | 1 |  |  |
| What is the location of the surgery?  PROMPT: If you can’t remember the address, the name of the street and/or borough will be fine. Or any major landmark nearby. |  |  | gpaddress |  |
| When you last used medical services, did you have a postal address? | No | 0 | postal |  |
|  | Yes | 1 |  |  |
| Do you remember the address? | No [🡪] | 0 | remember |  |
|  | Yes | 1 |  |  |
| What was the street, city and postcode? Type in as much as you remember. |  |  | address |  |
| Part 4 – Postscript |  |  |  |  |
| Our research team is interested in talking to a few people who have completed this questionnaire. We want to have an open-ended conversation experiences with accessing health care.  We ask for about 45 minutes of your time, and we’d offer £10 as a token of appreciation.  Can someone from King’s College London get in touch to tell you more? | No |  | qualpermission |  |
|  | Yes |  |  |  |
| [If yes]  What is the best way to contact you?  [enter email address, or phone number, or key worker contact details] |  |  | qualcontact |  |
| We have come to the end of the questionnaire. |  |  |  |  |
| Here is an envelope with £10. Thank you for participating in the study. |  |  |  |  |
| Part 5 - Post-questionnaire documentation |  |  |  |  |
| Were there any unexpected / unusual events to report? | No | 0 | anyevent |  |
|  | Yes | 1 |  |  |
| What were the unexpected /unusual event(s)? | Harm to self | 1 | event |  |
|  | Harm to child | 2 |  |  |
|  | Participant needs to leave | 3 |  |  |
|  | Participant withdraws consent | 4 |  |  |
|  | Participant falls asleep | 5 |  |  |
|  | Other people interrupt / get too close | 6 |  |  |
|  | Participant has a medical issue | 7 |  |  |
|  | Participant starts to drink / use drugs | 8 |  |  |
|  | Participant experiences distress from the questionnaire | 9 |  |  |
|  | Participant gets distracted / disinterested | 10 |  |  |
|  | Confusing question | 11 |  |  |
|  | Harassment | 12 |  |  |
|  | Tablet fails | 13 |  |  |
|  | Bad internet/phone connection | 14 |  |  |
|  | Other | 77 |  |  |
| Provide details about the event. |  |  | eventdetail |  |
| The interview is complete. Swipe right, and click 'Save Form and Exit'. | | | |  |
