# Supplementary material for: Peer advocacy and access to hospital care for people who are homeless in London, UK, 2019–2023: a cohort study
Source: BMJ Open. 2026 Jul 16;16(7):e107422. doi: 10.1136/bmjopen-2025-107422 (PMC13384207; doi:10.1136/bmjopen-2025-107422)
Supplement: online supplemental file 2 [file bmjopen-16-7-s002.docx]

**Supplementary Table 1 (S1): Characteristics of participants by linkage to Hospital Episode Statistics and by intervention arm among those successfully linked**

|  | **All participants**  **(n=311)** | | **Linked participants (n=229)** | | **Std Differences** | |
| --- | --- | --- | --- | --- | --- | --- |
|  | **Unlinked** | **Linked** | **Non-clients** | **Clients** | **Unweighted** | **Weighted** |
| **Variable** | **n (col %)** | **n (col%)** | **n (col %)** | **n (col %)** |  |  |
| Total | 82 (26.4) | 229 (73.6) | 100 (63.3) | 129 (84.3) |  |  |
| Age, years median (IQR) | 45 (40-53) | 49 (40-57) | 44 (35-55.5) | 51 (45-59) | 0.57 | 0.57 |
| Gender** |  |  |  |  |  |  |
| Male | 68 (82.9) | 176 (76.9) | 76 (76.0) | 98 (76.0) |  |  |
| Female | 14 (17.1) | 53(23.1) | 22 (22.0) | 30 (23.3) | 0.01 | -0.09 |
| Ethnicity |  |  |  |  |  |  |
| White | 45 (54.9) | 158 (69.0) | 64 (64.0) | 94 (72.9) |  |  |
| Black | 9 (11.0) | 32 (14.0) | 21 (21.0) | 11 (8.5) | -0.36 | -0.07 |
| Asian, other, multiple, refuse | 28 (34.2) | 39(17.0) | 6 (6.0) | 8 (6.2) | 0.10 | -0.03 |
| Citizenship |  |  |  |  |  |  |
| Non-UK | 25 (30.5) | 43 (18.8) | 23 (23.0) | 20 (15.5) |  |  |
| UK | 57 (69.5) | 186 (81.2) | 77 (77.0) | 109 (84.5) | 0.19 | -0.02 |
| Sexual orientation |  |  |  |  |  |  |
| Heterosexual | 72 (87.8.7) | 198 (86.5) | 82 (82.0) | 116 (89.9) |  |  |
| Gay, lesbian, bisexual, other, refuse | 10 (12.2) | 31 (13.5) | 18 (18.0) | 13 (10.1) | -0.23 | -0.03 |
| Education |  |  |  |  |  |  |
| Less than secondary | 15 (18.3) | 25 (10.9) | 10 (10.2) | 15 (11.7) |  |  |
| Secondary | 38 (46.3) | 127 (55.5) | 49 (50.0) | 75 (58.6) | 0.14 | 0.02 |
| More than secondary | 29 (35.4) | 77 (33.6) | 39 (39.8) | 38 (29.7) | -0.21 | 0.04 |
| Past experience of multiple exclusion |  |  |  |  |  |  |
| Begged | 27 (32.9) | 101 (44.1) | 35 (35.0) | 66 (51.2) | 0.31 | 0.11 |
| Injected drugs | 16 (19.5) | 74 (32.3) | 23 (23.0) | 51 (39.5) | 0.36 | 0.03 |
| Incarcerated | 35 (42.7) | 120 (52.4) | 43 (43.0) | 77 (59.7) | 0.33 | 0.05 |
| Years since first homeless median (IQR) | 9 (4-22) | 12 (5-28) | 9.5 (4-16) | 21 (7-33) | 0.77 | 0.20 |
| Sleeping location, last night |  |  |  |  |  |  |
| Unstable | 22 (26.8) | 39 (17.0) | 21 (21.0) | 18 (14.0) | 0.18 | -0.01 |
| Stable | 60 (73.2) | 190 (83.0) | 79 (79.0) | 111 (86.0) |  |  |
| Transport is a barrier to health care |  |  |  |  |  |  |
| Yes | 53 (64.6) | 135 (59.0) | 51 (51.0) | 84 (65.1) | 0.30 | 0.05 |
| No | 29 (35.4) | 94 (41.0) | 49 (49.0) | 45 (34.9) |  |  |
| Used heroin, 12 months |  |  |  |  |  |  |
| No | 68 (82.9) | 152 (66.4) | 74 (74.0) | 78 (60.5) | 0.31 | 0.00 |
| Yes | 14 (17.1) | 77 (33.6) | 26 (26.0) | 51 (39.5) |  |  |
| PHQ4 score category |  |  |  |  |  |  |
| 0-5 (Green Flag) | 30 (37.5) | 82 (36.6) | 34 (34.4) | 48 (38.4) |  |  |
| 6-8 (Yellow flag) | 19 (23.7) | 57 (25.5) | 24 (24.2) | 33 (26.4) | 0.05 | 0.003 |
| 9-12 (Red flag) | 31 (38.8) | 85 (37.9) | 41 (41.4) | 44 (35.2) | -0.13 | 0.016 |

ⱡ measured through PHQ4 ** Participants (<5) who selected another gender option were recoded to male or female using their given name to minimise risk of identification. Unstable housing = rough, sofa surf, B&B, emergency shelter; Stable housing = hostel, supported housing, own tenancy, B&B, Bed & Breakfast; DNA, Did-not-attend; HHPA, Homeless Health Peer Advocacy
