# Supplementary material for: Peer advocacy and access to hospital care for people who are homeless in London, UK, 2019–2023: a cohort study
Source: BMJ Open. 2026 Jul 16;16(7):e107422. doi: 10.1136/bmjopen-2025-107422 (PMC13384207; doi:10.1136/bmjopen-2025-107422)
Supplement: Supplementary file 3 [file bmjopen-2025-107422.reviewer_comments.pdf]

PEER REVIEW HISTORY

BMJ Open publishes all reviews undertaken for accepted manuscripts. Reviewers are asked to complete a checklist review form and are provided with free text boxes to elaborate on their assessment. These free text comments are reproduced below.

ARTICLE DETAILS

Title (Provisional)

Peer advocacy and access to hospital care for people who are homeless in London, United Kingdom 2019-2023: a cohort study

Authors

Platt, Lucy; Guise, Andy; Hosseini, Paniz; Bowgett, Kate; Murphy, Martin; Cudjoe, Mani; Annand, PJ; Hudson, Michael 'Spike'; Yankah, Maame Esi D.; Legood, Rosa; Menezes, Dee; Aldridge, Robert W; Hayward, Andrew; Luchenski, Serena; Williamson, Elizabeth; Rathod, Sujit D

VERSION 1 - REVIEW

**Reviewer**	**1**
**Name**	**Parkes, Tessa**
**Affiliation**	**University of Stirling, Faculty of Social Sciences**
**Date**	**14-Oct-2025**
**COI**	**None**

Overall comments

Thank you for inviting me to review this important paper. As the authors suggest, this is an area which needs more research in order to draw valid conclusions concerning the benefits of peer workers in health and wider systems of care for people that mainstream services find it harder to reach/engage with. It is also a very challenging environment to undertake research within. I therefore commend the author team for having the vision and ambition to design and implement a bold study that aims to quantify the effectiveness of peer advocacy for this under-served client group using a robust outcome measure - access to hospital care via scheduled outpatient appointments. I would also like to note how difficult it must have been to deal with COVID-19 challenges, on top of the usual demands of conducting high quality research in this field. The author team have shown resilience in managing these challenges and successfully concluding this study. I would finally like to highlight in these opening comments the fact that the author team includes those working within the third sector partner organisation, Groundswell. Including organisational partners as authors is not as common as it should be.

As a researcher working in the UK, and in this particular field (peer workers and homelessness), I will be referring to work my team has undertaken of relevance in this review. This is not to suggest that this work be cited necessarily but because I believe that it is important in general that researchers take due account of related work undertaken within the same country. In this case it is work funded by the same organisation.

I need to also state I am not an expert in cohort design/methods, nor on the statistical analysis used, so I am limiting my review feedback to areas I have expertise in only expecting editors to ensure that all elements of this paper have had rigorous scrutiny. I hope that my comments are all helpful and constructive for the author team.

Abstract

Spell out HHPA in the body of the abstract

I think you need to add the word volunteer to trained advocates (below). So… volunteer trained advocates.

Intervention: Peer advocacy is the provision of support by trained advocates with lived experience of homelessness to individuals to overcome barriers to accessing health services.

This is to differentiate the intervention from those where the peer support is provided by paid workers as I believe this is important to clarify for readers.

PHQ4 is patient health questionnaire not public health.

Summary

Yes, I had wondered whether assuming non-attendance among unlinked individuals was appropriate. Perhaps the authors could say more about ways they tried to check this dataset completeness in advance of the study? Perhaps the authors could comment on what work had been done to examine this dataset prior to use?

It would be helpful for readers to know why the study was still embarked upon during the COVID-19 period, given the likelihood that this would become a really challenging factor in

the way described given the impact of the pandemic on services for people experiencing homelessness. Was there pressure from funders for example?

Introduction

Page 4 – typo – should be well not week

This represents avoidable ill-health and distress, as week as considerable costs to the health system: healthcare costs for homeless individuals are estimated to be eight times higher than those of the general population.(7)

Page 4 (according to bottom of page numbering)

References to describe peer work in the field of drugs/substance use. There is UK NIHR funded work in this area that would be appropriate to review to see if it is of relevance to this introduction given it is focused on the clients experiencing both homelessness and substance use and contains outcomes data. See https://fundingawards.nihr.ac.uk/award/16/153/14

Peer advocacy is not, in my view, the same as peer support but it is used interchangeably in this section. I would suggest that a definition be provided for the type of peer advocacy used in the intervention and this terminology retained through the paper. The title and objectives state the intervention is peer advocacy specifically. Peer support is far more general. Reading on in the paper I see this definition is provided later so I would encourage the authors to move this earlier for clarity.

The project was overseen by a steering group consisting of people working in the homeless sector and with lived experience of homelessness to guide the design and interpretation of findings.(20)

Does the above refer to study design or intervention design or both? It would be helpful for the authors to describe exactly how the study and intervention design had input from people with relevant lived / living experience. It would also be common to have a separate PPI group as well as study steering group on an NIHR study. Was there one group that undertook both these functions combined? More information would be helpful here for readers interested in how to do this work well.

Page 5 (numbered at bottom of page not top which is 6, I will use the page numbers at the bottom of the page in my review comments)

Please be clear when describing the peer workers that they are volunteers not paid workers. Please also note if there are contracts in place for the work undertaken. It would be helpful to know whether safeguards were put in place to ensure that the peer advocates worked to their stated responsibilities in addition to training and supervision? Was there a protocol regarding confidentiality and breaking of it for safeguarding and how about managing risk of suicide? How about fidelity?

In the final paragraph under intervention and impact of COVID-19 please consider adding the additional information on the impact of COVID-19 to be clear. Perhaps after the words

During the recruitment period (July to December 2021) WHICH WAS STILL IMPACTED BY THE COVID-19 PANDEMIC,

Recruitment section – please can more information be provided on the processes of recruiting clients and whether this followed the published study protocol and any variations? It would be helpful to refer to the protocol at this stage. Given the necessary brevity of this results paper and the existence of the published protocol which I found very helpful to read alongside, perhaps the authors could signal to readers any areas where the final study did not follow the published protocol for transparency?

Typo line 35, page 5 – they were working, not there were working

Page 6

Top of page 6 – with regard to the £10 provided – was this in cash or vouchers?

I suggest to add magazine after Pavement and provide a reference.

I do not see the full questionnaire in the list of supplementary files so this needs to be provided for transparency.

We measured anxiety and depression using the Patient Health Questionnaire (PHQ)-

4 (29) categorising participants with scores of 0-5 as low risk, 6-8 as moderate risk and 9-12 as

severe risk.(30)

I suggest to be clear – low / moderate / severe risk of what? My understanding of PHQ4 is it is a measure of categories of psychological distress (none, mild, moderate, severe), rather than calculating specific risks. Same in results section – perhaps reword to avoid making it sound like it is ascertaining risk – it is really about the presence or absence of a psychological condition. If this is a point about suicide risk, please make that clear including what processes were followed by the team for such high risk individuals where a person scored as severe on the PHQ4. I do not see anything about interventions for those deemed high risk of suicide which the PHQ would indicate.

Missing data section page 6. Please describe what function did the 12 months prior / pre serve?

Page 7

311 was less than the protocol minimum sample size which was 386. Please provide a reason for this and discuss the impact regarding the sample size and power in the paper. I see this is in the limitations later in the paper but my view would be that this should be noted earlier in results or methods, given its importance for the study.

Table 1 – close brackets needed for total (n+311)

Discussion

In the introduction of the paper, inpatient visits are deemed to be preventable and not ideal - and it is stated that they have additional personal and healthcare costs i.e that these are unwanted impacts of not having early scheduled / preventive health interventions in a clients' care journey. Please can the authors discuss whether this could be seen as an unintended negative impact from the study intervention? Or is it deemed better that the clients were at least getting care as opposed to not getting care? It is an interesting and important discussion point to potentially elaborate on a bit more.

As noted in my comments above, the information under limitations could have been noted earlier in the methods section i.e. the reasons for a smaller sample and change to the inclusion criteria that was written in the protocol and then returned to in limitations to discuss the impact on the study findings.

Connected to this, I wonder if one of the reasons for lower numbers of participants was because some were concerned about the data linkage element? This is something we have found in our own work. It would be helpful to know the number of clients who refused involvement regarding involvement on the basis of fears around data linkage, if the authors recorded this, as this is an important finding for other researchers working with this client group.

**Reviewer**	**2**
**Name**	**Nisenbaum, Rosane**
**Affiliation**	**St. Michael's Hospital, Centre for Research on Inner City Health**
**Date**	**02-Dec-2025**
**COI**	**None**

Thank you to the authors for the intervention they designed and conducted. Innovative interventions for individuals experiencing homeless are very much needed, and these interventions cannot be implemented without collaboration of researchers with peers with lived experience. This is certainly one strength of their study. Below, I provide some feedback that I hope will be useful for further analyses and reporting of their data.

Abstract

-Please ensure typos, punctuation and misspelling are corrected in this section and throughout the manuscript. Acronyms need not be defined more than once (HES)

-On the other hand, what does HHPA, A&E stand for? Please make sure all acronyms are defined before using them in the Abstract and throughout the manuscript

-May I suggest not using DNA as an acronym, due to its meaning in genetics?

-The objective is related to hospital use, however the primary outcome is related to outpatient appointment. Please reconcile and edit for consistency.

-In principle, “do not attending” is a binary outcome, and it is, at this point, modelled using the Poisson distribution because of differential person-time. On the other hand, the number of inpatient admissions, accidents and emergency visits are count outcomes, and should have been analyzed using the Poisson or negative binomial models.

-It was not until I read the Conclusion that I realized the work aimed at addressing the COVID-19 pandemic. This should have been clearly stated at the beginning

Article Summary

-Terminology needs to be consistent throughout the manuscript. Abstract refers to clients vs non-clients, Summary refers to intervention and comparison/usual care groups. Please integrate

Introduction

-May I suggest that the research objective of the study be included in the last section of Introduction? As written if reads more like 'Aim' than the specific 'Research Objective'.

Methods

-Information is repeated, please consolidate

-What is the Pavement? (info needed for readers not in the UK or Europe)

-This reviewer interprets that the outcome listed in the Abstract Objective (hospital use) does not relate to the primary outcome (outpatient visits) but to secondary outcomes. Please re-articulate.

-How does one reconcile the homelessness definition with "own tenancy in the previous night"? Perhaps residence in the previous night should also be a confounder to the relationship between intervention and outcomes?

-In sensitivity analysis, the missing data assumptions for those who were not linked to any of the databases are very strong. Can the authors justify?

-As stated above, linear models should not have been used to analyze count outcomes.

-Isn't the number of not attended appointments = number of scheduled appointment minus the number of attended appointments? Why model both?

-Perhaps any extra analyses should be considered as Exploratory (e.g. type of engagement: 0-1 vs 2 or more)

-What interaction terms were included in the propensity score model? All variables should have been listed in Methods

-The relative probability was later interpreted as risk from the rate ratio. Kindly integrate terminology.

Results;

-What would explain the very low response rate among approached clients, but not non-clients?

-Subscripts or abbreviations should be explained Table footnotes

-Please double check percentages in Tables since there are a few mistakes

-Shouldn't the denominator in Table 2 for post-intervention be the number of participants linked at least once? According to methods "Those who were not identified in any of the databases were assumed to be unlinked and missing."

-Table 2 is difficult to interpret because denominators are not constant across the 6 databases.

-In Table 3, it is not surprising that medians are zero, since these are probably zero-inflated outcomes (most participants did note experience the outcome in the 12 months post-intervention). Clinically relevant categories could be more informative

- As stated above, Poisson or negative binomial models should be applied to count secondary outcomes.

Discussion

Too many loose ends, unfortunately, rendering the manuscript difficult to follow.

- Interpretation is compromised.

VERSION 1 - AUTHOR RESPONSE

Reviewer: 1
Prof. Tessa Parkes, University of Stirling
Comments to the Author:
Overall comments

Thank you for inviting me to review this important paper. As the authors suggest, this is an area which needs more research in order to draw valid conclusions concerning the benefits of peer workers in health and wider systems of care for people that mainstream services find it harder to reach/engage with. It is also a very challenging environment to undertake research within. I therefore commend the author team for having the vision and ambition to design and implement a bold study that aims to quantify the effectiveness of peer advocacy for this under-served client group using a robust outcome measure - access to hospital care via scheduled outpatient appointments. I would also like to note how difficult it must have been to deal with COVID-19 challenges, on top of the usual demands of conducting high quality research in this field. The author team have shown resilience in managing these challenges and successfully concluding this study. I would finally like to highlight in these opening comments the fact that the author team includes those working within the third sector partner organisation, Groundswell. Including organisational partners as authors is not as common as it should be.

As a researcher working in the UK, and in this particular field (peer workers and homelessness), I will be referring to work my team has undertaken of relevance in this review. This is not to suggest that this work be cited necessarily but because I believe that it is important in general that researchers take due account of related work undertaken within the same country. In this case it is work funded by the same organisation.

I need to also state I am not an expert in cohort design/methods, nor on the statistical analysis used, so I am limiting my review feedback to areas I have expertise in only expecting editors to ensure that all elements of this paper have had rigorous scrutiny. I hope that my comments are all helpful and constructive for the author team.

Abstract

Spell out HHPA in the body of the abstract
I think you need to add the word volunteer to trained advocates (below). So… volunteer trained advocates.
Intervention: Peer advocacy is the provision of support by trained advocates with lived experience of homelessness to individuals to overcome barriers to accessing health services. This is to differentiate the intervention from those where the peer support is provided by paid workers as I believe this is important to clarify for readers.
PHQ4 is patient health questionnaire not public health.

Response: We thank the reviewer for highlighting the errors. These have been addressed.

Summary

Yes, I had wondered whether assuming non-attendance among unlinked individuals was appropriate. Perhaps the authors could say more about ways they tried to check this dataset completeness in advance of the study? Perhaps the authors could comment on what work had been done to examine this dataset prior to use?

Response: Prior research had been conducted by co-authors demonstrating the feasibility of measuring hospital discharges among people who are homeless via data linkage to hospital episode statistics.(1) Formative work had been conducted to inform analyses to estimate health needs as prevention opportunities for people who are homeless and scale of hospital admissions that has subsequently been published. (2, 3) A small scale evaluation of the HHPA programme also demonstrated that data linkage was feasible and focus groups with people who are homeless demonstrated acceptability of data linkage to the population. (4, 5)

It would be helpful for readers to know why the study was still embarked upon during the COVID-19 period, given the likelihood that this would become a really challenging factor in the way described given the impact of the pandemic on services for people experiencing homelessness. Was there pressure from funders for example?

Response: Recruitment had started prior to the first lockdown. We restarted recruitment in July 2021 when HHPA had been working again. We held lengthy discussion with our steering group and with Groundswell about whether we should continue the evaluation, but it was agreed that HHPA was running sufficiently to justify continuing. We explored in the linked qualitative work how it adapted to the pandemic. We agreed that while there were changes to our outcomes, these changes would affect both the client and non-client group and therefore not differentially bias findings. This has been explained on page 5 'Intervention and impact of COVID-19'.

Commented [SDR1]: Note reference to "steering group" below.

Commented [LP2]: Add to this paragraph.

Introduction

Page 4 – typo – should be well not week

This represents avoidable ill-health and distress, as week as considerable costs to the health system: healthcare costs for homeless individuals are estimated to be eight times higher than those of the general population.(7)

Corrected

Page 4 (according to bottom of page numbering)
References to describe peer work in the field of drugs/substance use. There is UK NIHR funded work in this area that would be appropriate to review to see if it is of relevance to this introduction given it is focused on the clients experiencing both homelessness and substance use and contains outcomes data. See https://fundingawards.nihr.ac.uk/award/16/153/14

Response: Thank you for bringing this to our attention. We agree that – reference to this work has been made.

Peer advocacy is not, in my view, the same as peer support but it is used interchangeably in this section. I would suggest that a definition be provided for the type of peer advocacy used in the intervention and this terminology retained through the paper. The title and objectives state the intervention is peer advocacy specifically. Peer support is far more general. Reading on in the paper I see this definition is provided later so I would encourage the authors to move this earlier for clarity.

Response: We thank the reviewer for highlighting this important point and agree that better specification of the type of intervention delivered by peers is necessary when describing the evidence. We have made it clear in the introduction where the evidence refers to peer advocacy or other types of peer support and what that consists of. We have made sure that we have consistently used the terminology of peer advocacy to describe the Homeless Health Peer Advocacy intervention. While we agree with the reviewer that there is a difference between more general support and advocacy. HHPA peers officially offer support for issues affecting clients' access to health care, for other issues they signpost the client to services. In reality peer advocates can end of lending support across varied aspects of their lives. We have discussed the implications of this on our findings in the discussion.

Commented [LP3]: Need to do this.

The project was overseen by a steering group consisting of people working in the homeless sector and with lived experience of homelessness to guide the design and interpretation of findings.(20)

Does the above refer to study design or intervention design or both? It would be helpful for the authors to describe exactly how the study and intervention design had input from people with relevant lived / living experience. It would also be common to have a separate PPI group as well as study steering group on an NIHR study. Was there one group that undertook both these functions combined? More information would be helpful here for readers interested in how to do this work well.

Response: We have now clarified that the steering group oversaw the evaluation and not the intervention. This is an important distinction and thank the reviewer for bringing this to our attention. We did not have a separate PPI group, but people with lived experience were

a core part of our steering group. We have also clarified that Groundswell, is a non-governmental organisation working alongside specialist homelessness services in London, of which two thirds of staff have lived experience of homelessness.

Commented [SDR4]: Note reference to "advisory group" above

Page 5 (numbered at bottom of page not top which is 6, I will use the page numbers at the bottom of the page in my review comments)

Please be clear when describing the peer workers that they are volunteers not paid workers. Please also note if there are contracts in place for the work undertaken. It would be helpful to know whether safeguards were put in place to ensure that the peer advocates worked to their stated responsibilities in addition to training and supervision? Was there a protocol regarding confidentiality and breaking of it for safeguarding and how about managing risk of suicide? How about fidelity?

Response: Under the intervention description section, we have added a note stating that peers are volunteers and reimbursed for expenses. We have also added some additional detail describing the training and support peers receive to manage their work, safeguarding issues of their clients and their own mental health. In brief, peers complete six weeks of training, followed by a month's shadowing, before they can work as peer advocates. Volunteers are trained in boundaries, risk assessment, safeguarding and dealing with difficult behaviour and all are DBS checked. Volunteers receive work supervision from their Line Manager and group and Clinical Supervision from an external counsellor to ensure that peers are conducting the work they are commissioned to do so and to provide emotional support. Groundswell salaried staff support the volunteer peer advocates in every step of the process, working as line managers and organising counselling support as needed. Their role is also to provide training, facilitate DBS checks, process bookings from referral agencies ensuring a timely and effective response.

In the final paragraph under intervention and impact of COVID-19 please consider adding the additional information on the impact of COVID-19 to be clear. Perhaps after the words During the recruitment period (July to December 2021) WHICH WAS STILL IMPACTED BY THE COVID-19 PANDEMIC,

Response: This has been added in.

Recruitment section – please can more information be provided on the processes of recruiting clients and whether this followed the published study protocol and any variations? It would be helpful to refer to the protocol at this stage. Given the necessary brevity of this results paper and the existence of the published protocol which I found very helpful to read alongside, perhaps the authors could signal to readers any areas where the final study did not follow the published protocol for transparency?

Response: We have added in additional detail on how clients were recruited and how this differed from the original plan.

Typo line 35, page 5 – they were working, not there were working

Page 6

Top of page 6 – with regard to the £10 provided – was this in cash or vouchers?

I suggest to add magazine after Pavement and provide a reference.

I do not see the full questionnaire in the list of supplementary files so this needs to be provided for transparency.

Response: This information on the nature of the reimbursement (cash), further explanation that the Pavement is a magazine and the supplementary information has been added.

We measured anxiety and depression using the Patient Health Questionnaire (PHQ)-4 (29) categorising participants with scores of 0-5 as low risk, 6-8 as moderate risk and 9-12 as severe risk.(30)

I suggest to be clear – low / moderate / severe risk of what? My understanding of PHQ4 is it is a measure of categories of psychological distress (none, mild, moderate, severe), rather than calculating specific risks. Same in results section – perhaps reword to avoid making it sound like it is ascertaining risk – it is really about the presence or absence of a psychological condition. If this is a point about suicide risk, please make that clear including what processes were followed by the team for such high risk individuals where a person scored as severe on the PHQ4. I do not see anything about interventions for those deemed high risk of suicide which the PHQ would indicate.

Response: This is a good point. We have clarified that the PHQ4 scale refers to symptoms of anxiety or depression and rewritten the results and methods to make this clearer. Our protocol specifies the safeguarding procedures in place should a participant make it clear they were at risk of suicide or harming themselves. The protocol also included guidance for interviewers to reflect on any issues that had arisen during the course of the interview and direct the participant to an appropriate service or a key worker.

Missing data section page 6. Please describe what function did the 12 months prior / pre serve?

Response: All analyses adjusted for hospital use (related to that specific outcome) for the 12 months prior to enrolment. Historic hospital use is a confounder in this study as it is a determinant of access to HHPA and a key predictor of current service use (6) We therefore present hospital use measures in the 12 months before enrolment in our descriptive analysis and then added in the relevant measure as a pre-specified confounder in regression models. (page 6)

Page 7

311 was less than the protocol minimum sample size which was 386. Please provide a reason for this and discuss the impact regarding the sample size and power in the paper. I see this is in the limitations later in the paper but my view would be that this should be noted earlier in

results or methods, given its importance for the study.

Response: During the second COVID-19 lockdown we developed a contingency plan to allow for the fact that recruitment into the survey might not be feasible at all. This design involved matching HHPA clients to HES data and then using codes indicating homelessness extracting data on hospital use from a sample recorded in HES without their consent. This process required ethical approval from the confidentiality advisory group, following a consultation with people who are homeless to assess acceptability of the approach to the broader community and devising strategies for raising awareness of the study so that people could opt out. During this process, we revised our sample size calculation. Instead of estimating our sample size based on the an average number of DNAs of 0.06 among our HHPA clients and 0.42 for non-clients we estimated that HPPA clients will have 15% of scheduled outpatient appointments classified as 'did not attend' in 12 months, compared to 35% for those in the comparison group. This equates to recruiting 97 participants for analysis in each group to detect this difference with 90% statistical power and two-sided alpha of 0.05. We have included this deviation from the protocol into the limitations section.

Table 1 – close brackets needed for total (n+311)
Response: Noted and corrected

Discussion

In the introduction of the paper, inpatient visits are deemed to be preventable and not ideal - and it is stated that they have additional personal and healthcare costs i.e that these are unwanted impacts of not having early scheduled / preventive health interventions in a clients' care journey. Please can the authors discuss whether this could be seen as an unintended negative impact from the study intervention? Or is it deemed better that the clients were at least getting care as opposed to not getting care? It is an interesting and important discussion point to potentially elaborate on a bit more.

Response: We thank the reviewer for highlighting this point. We agree that the effect of HHPA on inpatient care is difficult to interpret. We have elaborated on this point to better clarify this ambiguity.

As noted in my comments above, the information under limitations could have been noted earlier in the methods section i.e. the reasons for a smaller sample and change to the inclusion criteria that was written in the protocol and then returned to in limitations to discuss the impact on the study findings.

Response: We agree with the reviewer's comment. We now describe how the methods deviated from the protocol in relation to the sample size and definition of the HHPA client group into the methods. We discuss the implications of these changes on the findings in the strengths and limitations section. We state that the smaller sample size due to few

outpatient appointments occurring (as a result of COVID-19) and fewer participants being linked to HES data particularly in the comparison group might have introduced type 2 errors and failure to accurately reject the null hypothesis. We also note that the change in our inclusion criteria for HHPA clients from newly enrolled clients to those who had used the service in the last 6 months, may also have introduced further bias into the analysis in two ways, firstly by introducing greater differences between intervention and comparison group at baseline and secondly reducing the magnitude of differences in non-attendances among HHPA clients between baseline and follow-up.

Connected to this, I wonder if one of the reasons for lower numbers of participants was because some were concerned about the data linkage element? This is something we have found in our own work. It would be helpful to know the number of clients who refused involvement regarding involvement on the basis of fears around data linkage, if the authors recorded this, as this is an important finding for other researchers working with this client group.

Response: With one exception, we did not observe concerns about data linkage during the recruitment or consent stages. The key barriers to recruitment in the comparison arm concerned lack of researcher access to some hostels and day centres, usually relating to staff turnover or non-response, and occasionally a desire to protect service users from distressing topics. The one exception concerns a participant who completed the questionnaire and soon thereafter contacted the research team to withdraw his consent, stating a concern about data linkage. After a short discussion we agreed to withdraw the participant from the study and delete his data. The population of homeless persons in London is more exposed to research activity and perhaps that is a reason we did not observe these concerns.

Reviewer: 2
Dr. Rosane Nisenbaum, St. Michael's Hospital, University of Toronto
Comments to the Author:
Thank you to the authors for the intervention they designed and conducted. Innovative interventions for individuals experiencing homeless are very much needed, and these interventions cannot be implemented without collaboration of researchers with peers with lived experience. This is certainly one strength of their study. Below, I provide some feedback that I hope will be useful for further analyses and reporting of their data.

Abstract
-Please ensure typos, punctuation and misspelling are corrected in this section and throughout the manuscript. Acronyms need not be defined more than once (HES)
-On the other hand, what does HHPA, A&E stand for? Please make sure all acronyms are defined before using them in the Abstract and throughout the manuscript
-May I suggest not using DNA as an acronym, due to its meaning in genetics?
-The objective is related to hospital use, however the primary outcome is related to outpatient appointment. Please reconcile and edit for consistency.

Response: Outpatient appointments in the context of the UK take place at hospitals. We have made this clearer under the description of the outcomes in the methods section. We have removed duplication of definitions of acronyms.

-In principle, “do not attending” is a binary outcome, and it is, at this point, modelled using the Poisson distribution because of differential person-time. On the other hand, the number of inpatient admissions, accidents and emergency visits are count outcomes, and should have been analyzed using the Poisson or negative binomial models.

Commented [LP5]: @Fizz need some input

-It was not until I read the Conclusion that I realized the work aimed at addressing the COVID-19 pandemic. This should have been clearly stated at the beginning

Response: The work did not aim to address the COVID-19 pandemic, but it was disrupted by the pandemic. We have tried to make this clearer in the methods in the paragraph 'Intervention and impact of COVID-19' (page 5) by including some additional context on how the pandemic affected the evaluation. Recruitment into the study initially began prior to the pandemic, in January 2020, then was halted due to the implementation of the first lockdown. Recruitment was restarted in July 2021 and completed in December 2021. During this period there were no lockdowns, but health services were still impacted by the COVID-19 pandemic.

Article Summary
-Terminology needs to be consistent throughout the manuscript. Abstract refers to clients vs non-clients, Summary refers to intervention and comparison/usual care groups. Please integrate

Response: We thank the reviewer for highlighting this and have reread the manuscript editing to improve consistency in language.

Introduction
-May I suggest that the research objective of the study be included in the last section of Introduction? As written if reads more like 'Aim' than the specific 'Research Objective'.

Response: We have added a specific research objective of the analysis presented in the paper.

Methods
-Information is repeated, please consolidate

Response: I have done this – but if you have any thoughts – please add.

-What is the Pavement? (info needed for readers not in the UK or Europe)

Response: We have clarified that the Pavement is a magazine for people who are homeless containing a directory of specialist services.

-This reviewer interprets that the outcome listed in the Abstract Objective (hospital use) does not relate to the primary outcome (outpatient visits) but to secondary outcomes. Please re-articulate.

Response: Outpatient appointments in the context of the UK take place at hospitals. We have made this clearer under the description of the outcomes in the methods section

-How does one reconcile the homelessness definition with "own tenancy in the previous night"? Perhaps residence in the previous night should also be a confounder to the relationship between intervention and outcomes?

Response: We have summarised our definition of homelessness at the start of the paper - it includes sleeping outside, in hostels, shelters and temporary accommodation, as well as residing in unsafe and inadequate housing (e.g. overcrowded conditions or settings involving domestic violence). This could include people with their own tenancy. Groundswell accepts clients with their own tenancy if they have been referred via a homeless service; this represents a small minority of their client group (n=27). Given the small numbers, and because housing status is embedded within our broader definition of stable housing, we did not treat residence in the previous night as a separate confounder. However, we acknowledge this as a potential source of heterogeneity.

-In sensitivity analysis, the missing data assumptions for those who were not linked to any of the databases are very strong. Can the authors justify?

-As stated above, linear models should not have been used to analyze count outcomes.

@Fizz can you help with this?

-Isn't the number of not attended appointments = number of scheduled appointment minus the number of attended appointments? Why model both?

-Perhaps any extra analyses should be considered as Exploratory (e.g. type of engagement: 0-1 vs 2 or more)

Response: We have renamed these secondary analyses as exploratory, given that they were not prespecified in the protocol we agree that exploratory is a more suitable label.

-What interaction terms were included in the propensity score model? All variables should have been listed in Methods

Response: The interaction terms were 1) ethnicity X historic count of outpatient DNA and 2) ethnicity X years since first became homeless. These have been added into the methods under 'Statistical Analyses'.

-The relative probability was later interpreted as risk from the rate ratio. Kindly integrate terminology.

We thank the reviewer for spotting this error and we have corrected the terminology to refer to relative rate (Statistical analysis page 7) and not relative probability.

Results;
-What would explain the very low response rate among approached clients, but not non-clients?

Response: All clients who were approached agreed to participate. However, not all active clients meeting our inclusion criteria were approached to participate. For non-clients we did not record the number of recruitment contacts and so are not able to report a participation rate.

-Subscripts or abbreviations should be explained Table footnotes

Response: This has been done

-Please double check percentages in Tables since there are a few mistakes

Response: We have checked the tables and they are correct. We have added a footnote to explain that percentages will not always come to 100% due non response or don't know responses.

-Shouldn't the denominator in Table 2 for post-intervention be the number of participants linked at least once? According to methods "Those who were not identified in any of the databases were assumed to be unlinked and missing."

Response: Table 2 is presented to describe the level of linkage by arm and by database among the participants who were recruited. For this table, the denominators are presented in the top row, with 153 participants recruited in the HHPA arm and 158 in the non-client arm. In the bottom row, we have the 129 and 100 of whom were linked participants, who comprise the sample for primary outcome analyses. The remainder are unlinked and assumed to be missing. Later in sensitivity analyses we assume these are not missing and impute 0 counts for these participants' outcomes. We have dropped the middle row called 'any appointment' pre-recruitment as this was redundant. We have clarified that the row summarizing any appointment refers to any appointment pre and post recruitment. We have also added int the denominators for each row to make this clearer.

-Table 2 is difficult to interpret because denominators are not constant across the 6 databases.

Response: In Table 2, the denominators are consistently 153 and 158. As described above we have added the denominators to every row to make this clear.

-In Table 3, it is not surprising that medians are zero, since these are probably zero-inflated outcomes (most participants did note experience the outcome in the 12 months post-intervention). Clinically relevant categories could be more informative

Response: Table 3 is of the 229 participants who were linked, i.e. who had at least one record in at least one database. Our aim in Table 3 is to summarise these participants' level of hospital activity including non-attendances. As so many of these outcome measures are highly skewed we presented the medians and IQRs. It is true that 0 counts are common, however we note the 75th percentiles are usually not zero, indicating a long-tail of high-frequency hospital use. For example, 25% of all linked participants had 2 or more inpatient admissions in the 12 months of follow up. We believe these counts are relevant for service users, social service providers, and clinical service providers.

Commented [LP6]: @Fizz can you advise on this response?

- As stated above, Poisson or negative binomial models should be applied to count secondary outcomes.

Commented [LP7]: Need input

Discussion
Too many loose ends, unfortunately, rendering the manuscript difficult to follow.
- Interpretation is compromised.

Response: We agree with the reviewer that the findings from the evaluation is not straight forward. This should not be a reason for not publishing findings of the evaluation.. . Need to add to this response.

Reviewer: 1
If you have selected 'Yes' above, please provide details of any competing interests.: Not applicable

Reviewer: 2
If you have selected 'Yes' above, please provide details of any competing interests.: not applicable

References

1. Blackburn RM, Hayward A, Cornes M, McKee M, Lewer D, Whiteford M, et al. Outcomes of specialist discharge coordination and intermediate care schemes for patients who are homeless: analysis protocol for a population-based historical cohort. BMJ Open. 2017;7(12):e019282.
2. Luchenski S, Aldridge R, Stevenson F, Tariq S, Hewett N, Hayward A. Hospital admissions for physical health and psychosocial adversity among people experiencing homelessness in England: a population-based retrospective cross-sectional study. Lancet. 2023;402 Suppl 1:S10.
3. Luchenski SA, Böhning D, Aldridge R, Stevenson F, Tariq S, Hayward AC. Estimating the scale of hospital admissions for people experiencing homelessness in England: a population-based multiple systems estimation study using national Hospital Episode Statistics. BMJ Public Health. 2025;3(2):e002978.
4. Finlayson S, Boelman V, Young R, Kwan A. Saving lives, saving money. How homeless health peer advocacy reduces health inequalities. London: Young Foundation 2016.
5. Luchenski S, Maguire N, Aldridge RW, Hayward A, Story A, Perri P, et al. What works in inclusion health: overview of effective interventions for marginalised and excluded populations. The Lancet. 2017.
6. Twomey CD, Baldwin DS, Hopfe M, Cieza A. A systematic review of the predictors of health service utilisation by adults with mental disorders in the UK. BMJ Open. 2015;5(7):e007575.

VERSION 2 - REVIEW

**Reviewer**	**1**
**Name**	**Parkes, Tessa**
**Affiliation**	**University of Stirling, Faculty of Social Sciences**
**Date**	**24-Apr-2026**
**COI**	

Thank you for the opportunity to review this revised manuscripts and addressing my comments. There are some typos / proofing issues but nothing substantial.
